# NF-kappaΒ-inducing kinase regulates stem cell phenotype in breast cancer

**DOI:** 10.1038/srep37340

**Published:** 2016-11-23

**Authors:** Karla Vazquez-Santillan, Jorge Melendez-Zajgla, Luis Enrique Jimenez-Hernandez, Javier Gaytan-Cervantes, Laura Muñoz-Galindo, Patricia Piña-Sanchez, Gustavo Martinez-Ruiz, Javier Torres, Patricia Garcia-Lopez, Carolina Gonzalez-Torres, Victor Ruiz, Federico Avila-Moreno, Marco Velasco-Velazquez, Mayra Perez-Tapia, Vilma Maldonado

**Affiliations:** 1 Instituto Nacional de Medicina Genómica (INMEGEN), México, 14610, México; 2 Unidad de Investigación Médica en Enfermedades Oncológicas (UIMEO), Hospital de Oncología IMSS, México; 3 Unidad de Investigación Médica en Enfermedades Infecciosas y Parasitarias (UMAE), Hospital de Pediatría, IMSS, México; 4 Instituto Nacional de Cancerología (INCAN), México; 5 Instituto Nacional de Enfermedades Respiratorias “Ismael Cosío Villegas” (INER), México; 6 Facultad de Estudios Superiores Iztacala, UNAM, México; 7 Facultad de Medicina UNAM, México; 8 Unidad de Desarrollo e Investigación en Bioprocesos (UDIBI) y Departamento de Inmunología, IPN, México.

## Abstract

Breast cancer stem cells (BCSCs) overexpress components of the Nuclear factor-kappa B (NF-κB) signaling cascade and consequently display high NF-κB activity levels. Breast cancer cell lines with high proportion of CSCs exhibit high NF-κB-inducing kinase (NIK) expression. The role of NIK in the phenotype of cancer stem cell regulation is poorly understood. Expression of NIK was analyzed by quantitative RT-PCR in BCSCs. NIK levels were manipulated through transfection of specific shRNAs or an expression vector. The effect of NIK in the cancer stem cell properties was assessed by mammosphere formation, mice xenografts and stem markers expression. BCSCs expressed higher levels of NIK and its inhibition through small hairpin (shRNA), reduced the expression of CSC markers and impaired clonogenicity and tumorigenesis. Genome-wide expression analyses suggested that NIK acts on ERK1/2 pathway to exert its activity. In addition, forced expression of NIK increased the BCSC population and enhanced breast cancer cell tumorigenicity. The *in vivo* relevance of these results is further supported by a tissue microarray of breast cancer samples in which we observed correlated expression of Aldehyde dehydrogenase (ALDH) and NIK protein. Our results support the essential involvement of NIK in BCSC phenotypic regulation via ERK1/2 and NF-κB.

Several reports have shown that tumors contain subpopulations of Cancer Stem Cells (CSCs) that can initiate and sustain tumor growth^1^. CSCs self-renew by generating unlimited copies and also give rise to mature non-stem cell progeny through differentiation, thus producing phenotypically different cells^1^,^2^. Breast cancer stem cells are classically defined by CD44 (Cluster of Differentiation antigen-44) positive and low or absent levels of CD24 (Cluster of Differentiation antigen-24) expression (CD44^+^/CD24^−/low^). Xenotransplant assays have revealed that as few as 100 cells with the CD44^+^/CD24^−/low^ phenotype can form tumors in immunodeficient mice^3^. Breast Cancer Stem Cells (BCSCs) also exhibit high levels of Wnt, Notch, Hedgehog, JAK/STAT and Nuclear factor-kappa B (NF-κB) activity; these pathways regulate self-renewal and differentiation processes^4^,^5^,^6^.

NF-κB refers to a family of transcription factors that control the expression of many genes related to immune responses, survival, proliferation, angiogenesis, and metastasis^7^. The NF-κB family consists of the following five transcription factors: RelA (p65), RelB, c-Rel, p100/p52, and p105/p50; these factors can homo or heterodimerize to allow DNA binding and activate transcription. Two main signaling pathways, the canonical, and non-canonical NF-κB pathways activate NF-κB; both pathways rely on signals that induce the phosphorylation and subsequent degradation of NF-κB inhibitors (IκB proteins). After degradation of NF-κB inhibitors, the NF-κB pathway is activated by translocation of NF-κB dimers. Canonical NF-κB pathway mainly induces the translocation of the p50:p65 dimer, while the non-canonical NF-κB pathway primarily triggers p52:RelB dimer translocation through NF-κB-inducing kinase (NIK)^8^,^9^.

NIK, a MAP kinase kinase kinase (MAP3K14) protein, is essential for the activation of the non-canonical NF-κB pathway because it phosphorylates IκB Kinase-α (IKKα) and participates in the processing of p100^10^. NIK also phosphorylates IκB Kinase-β (IKKβ) and activates canonical NF-κB pathway^11^. NIK is involved in processes such as cell differentiation, development, and embryogenesis; in the latter, NIK appears to play a role in pluripotent embryonic stem cell maintenance^12^. These activities of NIK support a potential role in the regulation of stem cell behavior^12^,^13^,^14^,^15^. In this regard, mutant mice with defects in the non-canonical NF-κB pathway, including NIK, display abnormalities in mammary gland development^16^,^17^,^18^.

NIK is frequently overexpressed in basal and claudin-low breast cancer cell lines, and its overexpression leads to constitutive NF-κB activation and proliferation in these tumor^19^,^20^,^21^. Basal and claudin-low carcinomas are mainly estrogen receptor (ER)-negative, progesterone receptor (PR)-negative, and human epidermal growth factor receptor 2 (HER2)-negative (triple negative). Triple negative tumors are more aggressive, have a poor prognosis, and contain higher proportions of BCSCs (CD44^+^/CD24^−/low^) than other tumor subtypes^22^,^23^. Recently, Zhang *et al.* observed that NIK-IKKα regulates HER2-induced mammary tumorigenesis by promoting the nuclear exclusion of p27/Kip1, thereby supporting the proliferation and expansion of BCSCs in a mouse tumorigenesis model^24^. In contrast to its role in breast cancer tumorigenesis, information about the role of NIK in CSC is limited. The aim of this project was to determine the role of NIK in the phenotype of BCSCs.

Here, we demonstrate that NIK is overexpressed in BCSCs isolated from MCF7 and MDA-MB-231 breast cancer cell lines. By disrupting NIK expression, we show that NIK inhibition affects the number of BCSCs and concomitantly reduces the expression levels of Aldehyde Dehydrogrenase-1A1 (ALDH1A1), NANOG, SOX2 (SRY-BOX2), and Octamer-Binding Transcription Factor (OCT4). In addition, we found that Aldehyde Dehydrogenase 1 (ALDH1) is co-expressed with NIK in tumor cells from patients with breast cancer. NIK inhibition impaired the ability of cells to grow tumors in immunodeficient mice. In support of these results, we also observed that NIK overexpression increased the proportion of CD44^+^/CD24^−/low^ cells and stem cell markers in MCF7 cells. Interestingly, microarray data revealed that NIK regulated stem cell-related genes through the Extracellular Signal-Regulates Kinases (ERK) pathway.

## Results

### Isolation of BCSCs Derived from Breast Cancer Cell Lines

**Figure 1:**
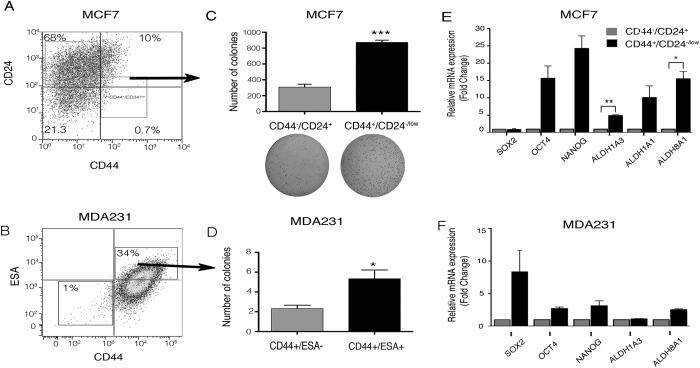
Characterization of Breast Cancer Stem Cell (BCSC) populations in MCF7 and MDA-MB-231 cell lines. (**A**) Fluorescence activated cell sorting (FACS) analysis of cell surface proteins showing proportions of Cancer stem cells (CD44^+^/CD24^low/−^) in breast cancer cell lines. (**B**) FACS analysis of CD44 and ESA showing proportions of CSCs in the MDA-MB-231 (triple negative) cell line. Three biological experiments were done for each cell line. (**C**) BCSCs (CD44^+^/CD24^low/−^) and non-BCSCs (CD44−/CD24+) populations isolated from MCF7 were tested for their clonogenic potential in soft agar assays (4,000 seeded cells). BCSCs showed increased clonogenic ability (n = 3, error bars are +/− s.e.m, *p < 0.05). (**D**) To confirm the clonogenic potential of MDA-MB-231 BCSCs (ESA^+^), limited dilution assays were performed. CD44^+^/ESA^+^ or CD44^−^/ESA^−^ cells colony numbers grown at low cell density. (n = 3, error bars are +/− s.e.m, *p < 0.05). (**E**) Cancer stem cell markers (NANOG, OCT4, SOX2, ALDH1A1, ALDH8A1, and ALDH1A3) expression was analyzed by Real time PCR (RT-qPCR) in MCF7 BCSC and non-BCSC populations. MCF7 BCSCs showed higher expression levels of CSC markers. (n = 3, error bars are +/− s.e.m, *p < 0.05). (**F**) Real Time-PCR analysis of CSC markers showed that CD44^+^/ESA^+^ cells expressed higher levels of SOX2, OCT4, NANOG and ALDH181. (n = 2, error bars are +/− s.e.m).

To isolate BCSCs, we used specific antibodies against surface markers (CD44, CD24 or Epithelial Cell Adhesion Molecule (ESA) in a luminal (MCF7) and a triple negative (MDA-MB-231) breast cancer cell line. BCSCs content varies greatly among breast cancer cell lines and breast carcinomas^25^,^26^,^27^. Triple negative tumors contain large numbers of CSCs while luminal breast tumors have lower stem cell contents^22^,^23^. Here, we demonstrated that MCF7 cell line has a low percentage of BCSCs (CD44^+^/CD24^−/low^ cells; 0.7–1.4%; Fig. 1A). Since MDA-MB-231 cell line exhibits low or null CD24 expression (data not shown), we used CD44 and ESA to detect the stem cell population (Fig. 1B). Thus, we determined that 34% of MDA-MB-231 cells were CD44+/ESA+ cells. In addition, we isolated a high ALDH activity subpopulation, since it was shown previously that this population presents phenotypic and functional characteristics of BCSC^28^. Thus, we determined that only 16% of MDA-MB-231 cells exhibit ALDH activity (Supplementary Fig. S1A,B).

BCSCs can initiate tumors and drive neoplastic proliferation. To verify the tumorigenic potential of BCSCs, we injected BCSCs and non-BCSCs into immunodeficient *nu/nu* mice. MCF7 cells with the CD44^+^/CD24^low/−^ phenotype were compared with CD44^−^/CD24^+^ cells. After 120 days, all mice inoculated with CD44^+^/CD24/^low/−^ MCF7 cells grew visible tumors, unlike CD44^−^/CD24^+^ cells (Supplementary Table S1). Sorted ESA^+^ MDA-MB-231 cells were able to form tumors within 90 days while the majority of mice inoculated with ESA^−^ did not form tumors (Supplementary Table S1).

To verify their clonogenic potential, isolated MCF7 BCSCs and non-BCSCs were seeded on soft agar plates. The number of colonies formed by CD44^+^/CD24^low/−^ cells was significantly higher than those formed by CD44^−^/CD24^+^ (818 vs. 236 colonies) (Fig. 1C). In addition, the clonogenic potential of sorted MDA-MB-231 BCSCs was evaluated in limited dilution assays, which revealed that the CD44^+^/ESA^−^ cells were more clonogenic than the CD44^−^/ESA^−^ cells (6 colonies vs. 2 colonies at low-density cell dilution) (Fig. 1D).

### BCSCs Express Higher Levels of NIK

CSCs express high levels of stem cell markers, which contribute to their self-renewal properties^28^,^29^. We found that isolated MCF7 CD44^+^/CD24^low/−^ cells exhibited higher expression levels of OCT4, NANOG, ALDH1A3, ALDH1A3 and ALDH8A1 compared to the CD44^−^/CD24^+^ subpopulation (Fig. 1E). BCSCs (CD44+/ESA+) derived from the MDA-MB-231 cell line, also expressed higher levels of some stem cell markers, including SOX2, OCT4, NANOG and ALDH8A1 compared to CD44−/ESA− cells (Fig. 1F).

**Figure 2:**
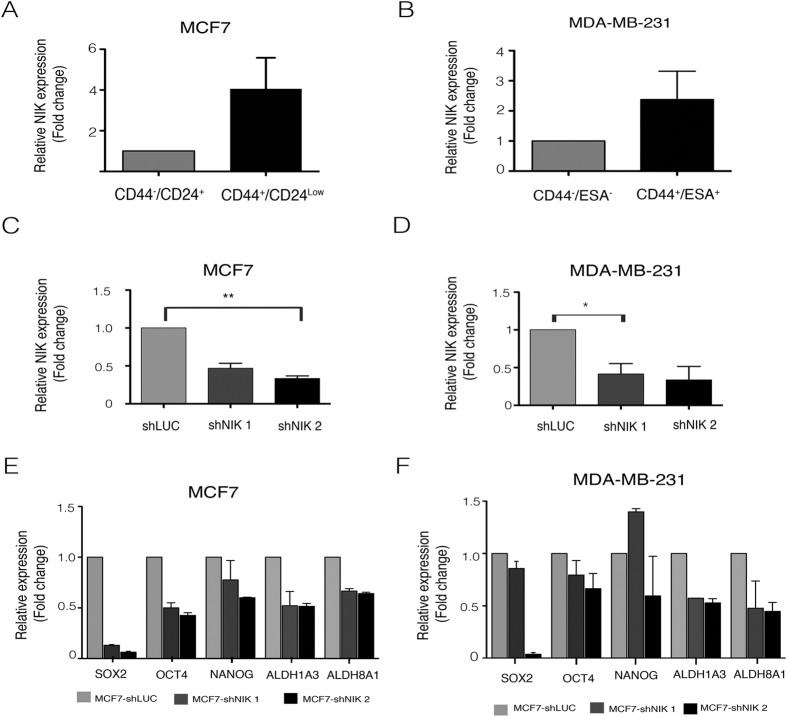
Nuclear factor-kappa B-inducing kinase (NIK) role in the regulation of Cancer stem cell (CSC) population of MCF7 and MDA-MB-231. (**A,B**) Breast cancer stem cell (BCSC) populations derived from MCF7 and MDA-MB-_231 are enriched in NIK. RT-qPCR analysis demonstrated that NIK is mainly expressed in the BCSC population of MCF7 (**A**) (n = 3, error bars are +/− s.e.m) and MDAMB231 (**B**) (n = 2, error bars are +/− s.e.m) cell lines. To knockdown NIK, two short hairpin RNA (shRNA) were used in MCF7 (**C**) and MDA-MB231 (**D**) cell lines. (n = 3, error bars are +/− s.e.m, *P < 0.05). (**E**) The impact of NIK expression over CSC markers levels was evaluated after stable inhibition of NIK in two independent MCF7 replicates. Real Time PCR analysis shows that NIK depletion affects the expression of CSC markers (SOX2, OCT4, ALDH1A3, ALDH8A1). (n = 2, error bars are +/− s.e.m). (**F**) Effect of NIK inhibition in two independent MDA-MB-231 stable transfected cells lines with shNIK1 or shNIK2. RT-qPCR analysis of markers expression after stable NIK inhibition in MDA-MB-231 cells. All Real Time PCR were normalized to TBP. The level of CSC markers or NIK was designated to 1 for MCF7 or MDA-MB-231 control cells (shLuc). (n = 2, error bars are +/− s.e.m).

NIK (also known as MAP3K14), a NF-κB activator, is upregulated in basal breast cancer cell lines^19^,^20^, which are enriched in CSCs^22^. For this reason, we examined the levels of this kinase in MDA-MB-231 (claudin low), SKBR3 (HER2+) and MCF7 (luminal) breast cancer cell lines and found that as expected, NIK is highly expressed in MDA-MB-231 cells and barely detectable in MCF7 cells (Supplementary Fig. S2). To determine if NIK expression in BCSCs was up-regulated, we examined its mRNA levels in a two cell lines. Figure 2A shows that MCF7 BCSCs expressed higher levels of NIK than the non-stem cell population (CD44^−^/CD24^+^). In addition, MDA-MB-231 BCSCs also exhibited higher levels of NIK when compared to CD44^−^/ESA^−^ cells (Fig. 2B); furthermore NIK is highly expressed in MDA-MB-231 cells with ALDH activity (Supplementary Fig. S1B). These results prompted us to investigate the role of NIK in the stem phenotype of breast cancer.

### NIK Inhibition Reduces the BCSC Population and Affects CSC Marker Expression

We next investigated the effect of NIK inhibition on the CSC phenotype. We generated stable MCF7 (MCF7-shNIK), MDA-MB-231 (MDA231-shNIK) and SKBR3 (SKBR3-shNIK) cell lines with two different small hairpin RNAs (shRNAs). RT-qPCR analysis showed that the two shRNAs efficiently reduced NIK expression in MCF7 (Fig. 2C), MDA-MB-231 (Fig. 2D) and SKBR3 cells (Supplementary Fig. S3A) in comparison with MCF7, MDA-MB-231 and SKBR3 control cells, respectively.

In MCF7, NIK deficiency reduced the expression of SOX2, OCT4, NANOG, ALDH1A3 and ALDH8A1. Similar results were obtained employing the second shRNA against NIK (Fig. 2E). In further support of these findings, NIK inhibition in MDA-MB-231 cells also reduced the expression of SOX2, OCT4, ALDH1A3 and ALDH8A1 (Fig. 2F). In addition, NIK deficiency also reduced the expression of SOX2, OCT4 and ALDH1A3 in SKBR3 cells (Supplementary Fig. S3B).

**Figure 3:**
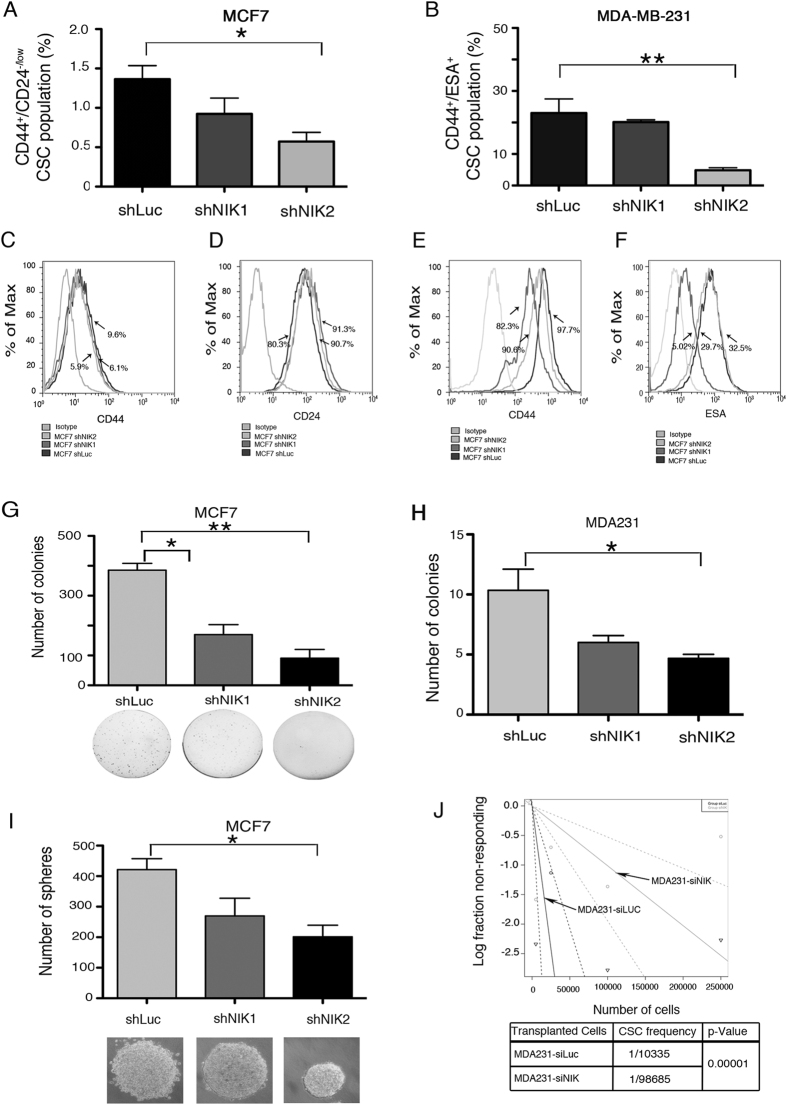
Nuclear factor-kappa-inducing kinase (NIK) deficiency impaired tumorigenic and clonogenic potential. (**A,B**) Frequency of CSCs in MCF7 (**A**) and MDA-MB-231 (**B**) depleted NIK cells compared to shLuc cells. (n = 3, error bars are s.e.m., *p < 0.05). (**C,D**) Flow cytometry analysis of CD44 (**C**) and CD24 (**D**) cell surface markers in NIK-deficient stable MCF7 cells. (**E,F**) Flow cytometry analysis of CD44 (**E**) and ESA (**F**) cell surface markers in NIK-deficient stable MDA-MB-231 cells and control cells respectively. Images represent an example of three independent experiments. (**G**) Soft agar clonogenic assays (4,000 seeded cells) reveals that stable NIK-deficient cells exhibit a reduced clonogenic ability of MCF7 cells. (n = 3, error bars are s.e.m, *p < 0.05. (**H**) Limited dilution assays showing colonies from three NIK depleted MDA-MB-231 cell lines grown at low cell density. (n = 3, error bars are s.e.m, *p < 0.05). (**I**) Number of spheres formed by three stable NIK-deficient MCF7 cell lines. (n = 3, error bars are s.e.m. *p < 0.05) (**J**) ELDA analysis showing cancer stem cell frequency in MDA-MB-231 deficient-NIK cells and MDA-MB-231 control cells xenotransplanted in nude mice. A total of 24 mice were xenotransplanted with 5000 (n = 5), 25000 (n = 6), 100000 (n = 8) or 250000 (n = 5) cells. MDA-MB-231 stable deficient-NIK cells were inoculated into the right flanks and MDA-MB-231 control cells in the left flank. Tumors were monitored by bioluminescence. The frequency of cancer stem cells was calculated using ELDA software.

BCSCs fraction was also reduced due to NIK inhibition in MCF7 and MDA-MB-231 cell lines. As shown in Fig. 3A,B, we found that there was a dramatic reduction in the BCSC population (CD44^+^/CD24^−^) in MCF7-shNIK1 and shNIK2. Expression of cell surface proteins was modified due to NIK inhibition (Fig. 3C–F). In MCF7 cell line, NIK inhibition provoked a slightly reduction in CD44 levels in comparison to control cells (Fig. 3C). On the contrary, NIK inhibition caused a 10% increase of cells expressing CD24, compared with the control cell line (Fig. 3D). To further support these results, we found that NIK inhibition also decreased the CD44 and ESA positive subpopulation in MDA-MB-231 cell line (Fig. 3E,F).

### NIK Depletion Impacts the Clonogenic and Tumorigenic Potential of Breast Cancer Cell Lines

Because our results show that NIK expression is associated with stem cell marker expression, we sought to determine its relevance in the cancer stem cell phenotype. Figure 3G shows that MCF7 deficient NIK cells formed significantly fewer colonies than MCF7-shLuc cells. The clonogenic potential of MDA-MB-231-shNIK cells was analyzed in limited dilution assays. MDA-MB-231-siNIK cells demonstrated reduced clonogenicity compared with MDA-MB-231-siLuc cells (Fig. 3H). Similar results were obtained with another shRNA against NIK. In addition, MCF7 depleted NIK cells formed lower numbers of mammospheres (Fig. 3I). Those results indicate that NIK depletion impairs the clonogenic potential of breast cancer cells.

To evaluate the tumorigenicity of NIK-depleted cells, we inoculated MDA231-shNIK cells carrying a luciferase reporter in nu/nu mice and analyzed the tumor burden six weeks later. The results showed that NIK inhibition reduced the tumor burden in most of the mice analyzed. Figure 3J show an ELDA analysis of inoculated mice which indicates that the CSC frequency is lower in MDA231-shNIK cells. These findings support the idea that NIK could be essential for tumorigenic abilities of BCSCs.

### Forced NIK Expression Increases CSC Marker Expression and BCSC Numbers

**Figure 4:**
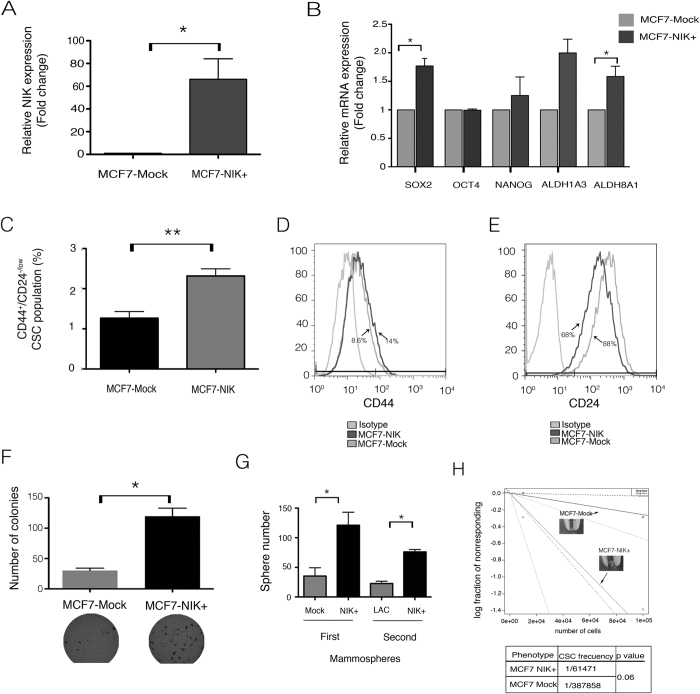
Forced expression of Nuclear factor-kappa B-inducing kinase (NIK) increases the Cancer stem cell fraction and increases tumorigenic and clonogenic potential. (**A**) RT-qPCR analysis showing increased NIK expression in relation to MCF7 control cells. (n = 3, error bars are s.e.m, *p < 0.05). (**B**) Expression of CSC markers in three stable NIK-overexpressing MCF7 cells. Levels of CSC markers were assessed by RT-qPCR assays and normalized to TBP. (n = 3, error bars are s.e.m., *p < 0.05). (**C**) Frequency of CSCs in MCF7 control and MCF7 over-expressing cells (n = 3, error bars are s.e.m., *p < 0.05). Results showed that forced expression of NIK increases the CSC fraction. (**D,E**) Flow cytometry analysis of CD44 (D) or CD24 (E) in MCF7 overexpressing NIK and MCF7 control cells. Images represent an example of three independent experiments. (**F**) Clonogenic assays showed that forced expression of NIK increase clonogenic ability. Experiments were performed in three independent stable cell clones. (n = 3, error bars are s.e.m., *p < 0.05). (**G**) Quantification of mammosphere number derived from MCF7 mock cells (MCF7-Mock) and NIK-overexpressing MCF7 cells (MCF7-NIK+) in both first and second passages. For mammosphere assay, 2.5 × 10^5^ cells/ml of each cell line were seeded. (n = 3, error bars are s.e.m., *p < 0.05). (**H**) A total of 8 mice were xenotransplanted with 100000 (n = 4) and 10000 (n = 4) MCF7 overexpressing cells or MCF7 control cells. MCF7 stable overexpressing-NIK cells were inoculated into the right flanks and MCF7 control cells in the left flank. Tumors were monitored each week during 120 days. The frequency of cancer stem cells was calculated using ELDA software.

To investigate the effects of NIK overexpression on the stem cell phenotype, we developed stable cell lines that overexpressed NIK (MCF7-NIK+) and (SKBR3-NIK+). qPCR analysis showed that MCF7-NIK+ and SKBR3+ cells expressed 60 fold (Fig. 4A) and 4 fold (Supplementary Fig. 3A) higher levels of NIK compared with MCF7-Mock and SKBR-Mock cells respectively. Our results showed that NIK overexpression increased the expression of some stem cell markers. In MFC7-NIK+ cells the most dramatic changes were the increase in ALDH1A3, ALDH8A1, and SOX2 (Fig. 4B). Overexpression of NIK in SKBR3 also increased the level of SOX2 and ALDH8A1 (Supplementary Fig. 3B).

Forced expression of NIK provokes an increment of CSC fraction in MCF7 cells (Fig. 4C). NIK overexpression slightly increases the expression of CD44 (Fig. 4D) and reduced CD24 expression levels (Fig. 4E), leading to an increase in the BCSC population (CD44^+^/CD24^low/−^).

To further investigate the clonogenic potential of MCF7-NIK+ cells, we performed soft-agar clonogenicity assays. Figure 4F shows that NIK overexpression increased the size and number of colonies formed by MCF7-NIK+ cells, when compared with MCF7-Mock+ cells. We also found that NIK-overexpressing MCF7 cancer cells developed a higher number of mammospheres and the sphere-forming potential is sustained even after a second passage (Fig. 4G). To further support these results, we evaluated the ability of SKBR3-NIK+ cells to form mammospheres, Supplementary Fig. 3C shows that NIK-overexpressing cells form a higher number of mammospheres than control cells.

To determine whether forced NIK expression promotes tumor growth, we inoculated *nu/nu* mice with MCF7-NIK+ or MCF7-Mock cells. NIK overexpression increased the number of tumors formed by MCF7 cells. ELDA analysis of inoculated mice showed that MCF7-NIK+ cells have a higher CSC frequency than MCF7-Mock cells (Fig. 4H). In addition, forced expression of NIK in SKBR3 cells also favored tumor formation and a higher CSC frequency (Supplementary Fig. S3D).

### NIK Expression Regulates NF-κB Molecules

**Figure 5:**
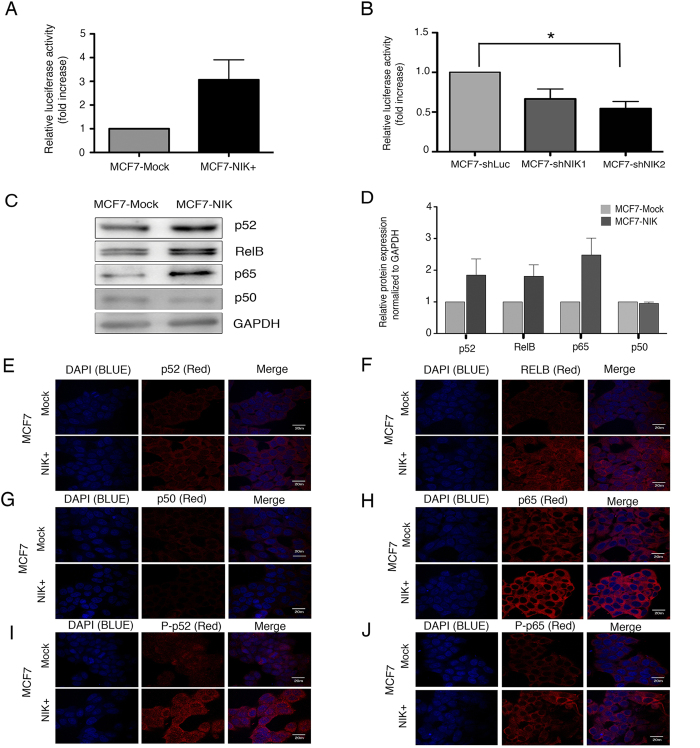
Nuclear factor-kappa B-inducing kinase (NIK) overexpression induces activation of canonical and non-canonical molecules . (**A**) Reporter gene assay showing the effects of NIK overexpression over NF-κB activity under basal conditions. Assays were performed in MCF7 cells transiently transfected with control or NIK expression vector. (n = 3, error bars are s.e.m.). (**B**) Reporter gene assay showing the effects of NIK depletion on NF-κB activity. Assays were performed in an MCF7 stable cell line bearing an NF-κB reporter vector. Stable cells were transiently transfected to inhibit NIK. NF-κB activity was calculated as fold difference between control and NIK-silenced cells. (n = 4, error bars are +/− s.e.m. *p < 0.05). (**C**) Western blot of NF-κB molecules in MCF7 enriched NIK and in MCF7 control cells. GAPDH was used as a loading control. (**D**) Densitometric scans from three independent assays were quantified, normalized to GAPDH and calculated as fold difference. (**E–J**) Immunofluorescence analysis of p52 (**E**), RelB (**F**), p50 (**G**), p65 (**H**), phospho-P52 (**I**) and phosphor-P65 (**J**) in MCF7 cells transiently overexpressing NIK and MCF7 control cells. Nuclear factor kappa B (NF-κB) proteins were stained with Cy3-conjugated secondary antibody and nuclei were stained with 4′-6-Diamidino-2-phenolindole (DAPI). Photographs are representative of three independent experiments. Images for immunofluorescence staining were taken using an x 63 oil lens.

NIK is an essential kinase that induces principally non-canonical NF-κB activation by phosphorylating p100 and IKKα^10^. Some reports have shown that NIK can also phosphorylate IKKβ and activate the canonical NF-κB pathway^11^,^30^. To verify NF-κB activation, we performed a reporter gene analysis in MCF7 cells. We observed that NIK overexpression dramatically increased NF-κB activation while NIK deficiency impaired NF-κB activation when compared to control cells (Fig. 5A,B).

It has been reported that NIK mediates the processing of p100 to p52 and promotes the nuclear translocation of p52:RelB dimer. In agreement with this, we found that NIK regulation resulted in modulation of nuclear p52. Supplementary Fig. S4A,B shows that MCF7-NIK+ cells presented a strong nuclear accumulation of p52, as opposed to MDA231-shNIK cells, in which, NIK inhibition resulted in a reduction of nuclear p52 (Supplementary Fig. S4C,D).

To determine which NF-κB molecules are involved in NIK-mediated regulation of CSC marker expression, we performed Western blotting and immunofluorescence analysis of key NF-κB proteins. Western blotting assays revealed that NIK increased mainly the expression of p52, RelB and RelA/p65 (Fig. 5C,D). Immunofluorescence analyses showed that NIK overexpression also increased the expression of p52 (Fig. 5E), RelB (Fig. 5F) and p65 (Fig. 5H). In addition, NIK overexpression also increased the phosphorylation of p100/p52 (Fig. 5I) and p65 (Fig. 5J). Interestingly, p50 levels were lower in MCF7-NIK+ cells (Fig. 5G).

### NIK Expression in Patients with Breast Cancer

**Figure 6:**
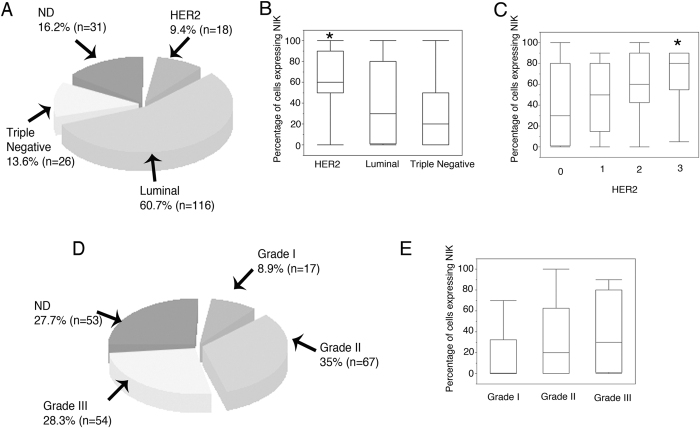
NIK is expressed mainly in the Human epithelial growth factor receptor (HER2) subtype. (**A**) Pie chart showing the percentage of HER2, Luminal, and Triple Negative breast cancer samples (n = 191). (**B**) Percentage of cells expressing NIK in HER2, Luminal, and Triple Negative breast tumors. NIK expression is higher in HER2 breast tumors (*p* = 0.01). (**C**) Percentage of cells expressing NIK in tumors with different expression levels of HER2 (0 = absent; 1 = weak; 2 = moderate; 3 = strong). NIK expression is higher in tumors with strong expression of HER2 (*p* = 0.005). (**D**) Pie chart showing the percentage of Grades I, II, and III breast cancer tissues (n = 191). (**E**) Percentage of cells expressing NIK in Grade I, II and III tumors. NIK expression levels increase in Grade III tumor (*p* = 0.07).

Finally, we determined NIK levels in 191 breast cancer tissue samples of which 60.7% were Luminal, 13.6% were triple negative and 9.4% were HER2 breast cancer subtype (Fig. 6A). To determine whether NIK expression is related to a particular breast cancer subtype, we examine NIK expression by immunohistochemistry, and we found that NIK expression was significantly higher in HER2+ breast carcinomas (Fig. 6B), and the expression of NIK positively correlated with HER2 expression levels (Fig. 6C). We also found that Triple Negative carcinomas exhibited the lowest expression of NIK, contrary to previous reports of breast cancer cell lines (Fig. 6B). Tumor Samples were also classified as grade 1, 2 and 3 (Fig. 6D). Interestingly, we observed a clear positive association between NIK and tumor grade (Fig. 6E).

**Figure 7:**
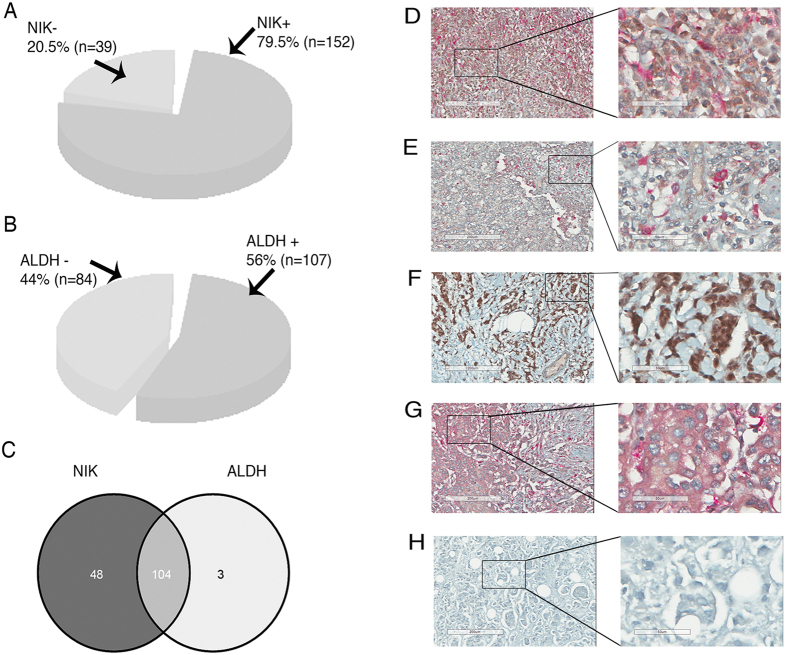
Immunohistochemistry analysis of NIK and Aldehyde dehydrogenase (ALDH) expression in breast carcinomas (*n* = 191). (**A**) Percentage of Nuclear factor-kappa B-inducing kinase (NIK)-positive breast tissues. (**B**) Percentage of ALDH positive breast tissues. (**C**) Venn diagram showing breast cancer tissues expressing both NIK and ALDH protein. (**D–H**) Immunohistochemistry of NIK and Aldehyde dehydrogenase (ALDH) expression. NIK-positive cells are brown based on DAB chromogen, and ALDH-positive cells are pink based on Fast Red chromogen. Nuclei are stained with hematoxylin (blue). The second column represents an enlargement of the area denoted by box in the adjacent left panel showing fine cellular details of immunohistochemistry. (**D**) Graph showing percentage of tissues having cellular colocalization in Breast Cancer samples expressing both NIK and ALDH. (**E**) Representation of breast tumors (*n* = 93) expressing ALDH and NIK in the same cells. (**F**) Immunohistochemistry of breast tumors showing NIK and ALDH expression in different cells (*n* = 10).

Immunohistochemistry analysis revealed that NIK was expressed in 79.5% (152) breast cancer tissues (Fig. 7A). To support our previous result showing that NIK and ALDH expression are correlated, we analyzed the co-localization of both proteins in these samples. ALDH was expressed in 56% of the studied cases (Fig. 7B), of which 97% also expressed NIK (Fig. 7C). Surprisingly, 89.4% of those tumors presented a cellular co-localization of both proteins as shown in Fig. 7D. Figure 7E represents tumors that express NIK and ALDH in different cells. Figure 7F–H represents tumors lacking either NIK or ALDH.

### NIK altered expression affects stem cell related genes

**Figure 8:**
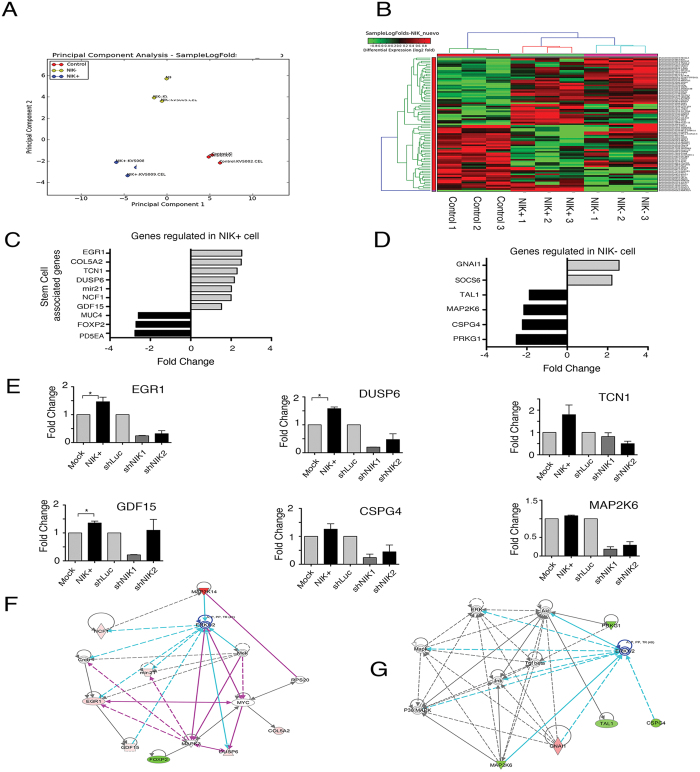
(**A**) Principal component analysis of gene expression profiles for high NIK-expressing MCF7 cells, low NIK-expressing MCF7 cells and MCF7 control cells. (**B**) Clustering diagram of samples and differentially expressed genes in MCF7 cells overexpressing NIK and NIK-deficient MCF7 cells. Log-Fold Changes of differentially expressed genes are depicted in a heat map, scaling from low (green) to high (red). Experiments were performed in three independent transiently-transfected MCF7 cell lines (**C**). Graph showing the fold change of stem cells-associated genes in high NIK-expressing cells and (**D**) low NIK-expressing cells. (**E**) RT-qPCR analysis of EGR1, TCN1, DUSP6, GDF15, CSPG4 and MAP2K6 in MCF7 overexpressing-NIK cells, MCF7 control cells, NIK-deficient MCF7 cells (shNIK1 and shNIK2) and MCF7 control cells (shLuc). All RT-qPCR were normalized to TBP. (n = 3, error bars are +/− s.d, p < 0.05). (**G**) IPA network of top gene networks from NIK-overexpressing cells. (**H**) IPA network of top gene networks from NIK-depleted cells. Note that ERK1/2 is a central node in both networks. Red color indicates induced genes and green color represents suppressed genes.

To elucidate the possible mechanism by which NIK affects the stem cell phenotype, we performed whole-genome microarray expression analysis of NIK-overexpressing, NIK-deficient, and MCF7 control cells. As expected, the principal component analysis divided the samples into three groups; high NIK expression (NIK+), low NIK expression (NIK−) and the control cells (Fig. 8A). We found significant gene expression differences in 79 genes in MCF7 overexpressing-NIK cells and 53 differential expressed genes in MCF7 deficient NIK cells (Supplementary Table S2 and S3 and Fig. 8B). Supporting the role of NIK in CSCs regulation, we found that most of the top regulated genes were involved in stem cell or EMT processes (Fig. 8C,D).

In order to validate the microarray data, seven genes were analyzed by real time RT-PCR. We based this selection on the fold change found or on their role in stem cell biology. All analyzed genes correlated well with the results obtained from microarrays (Fig. 8E). As expected, we observed that upregulated genes (EGR1. TCN1. DUSP6, GDF15) in the samples with high NIK expression were also downregulated in MCF7 depleted-NIK cells (Fig. 8E).

Ingenuity Pathway Analysis (IPA) of MCF7 cells overexpressing NIK showed concordant enriched signaling networks, including cellular growth and proliferation, cell death and survival, cellular movement and hematological system development. These results indicate that NIK disruption could impair the proliferation, survival, and migration of cancer stem cells. Our microarray results suggest that NIK could activate ERK1/2 pathway to regulate several molecules involved in stem cell modulation such as EGR1, NCF1, COL5A2 and GDF15) (Fig. 8F). In addition, our results showed that NIK deficiency down-regulated the expression of stem cell-associated genes such as MAP2K6, CSPG4, and TAL1, probably by the MAPK and ERK1/2 pathway (Fig. 8G). Data analysis of deregulated genes in MCF7 cells with high NIK expression revealed that ERK could be one of the key pathways that contribute to regulate the stem cell phenotype.

### NIK regulates stemness through ERK activation

**Figure 9:**
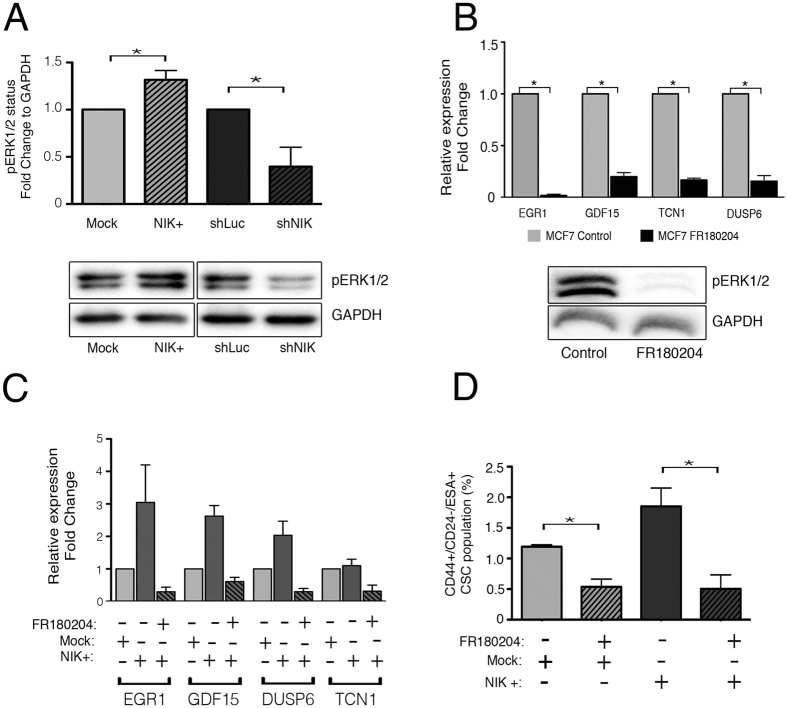
NIK regulates target genes through ERK pathway. (**A**) Western Blot analysis of phospho-ERK1/2 in MCF7 transiently expressing NIK, control vector or NIK knock-down cells. (n = 3, error bars are s.d., p* < 0.03). (**B**) Relative expression of target genes in MCF7 cells treated with 30 uM FR180204 or an equivalent amount of DMSO during 24 hrs. The lower image represents the effect of FR180204 on ERK1/2 inhibition. (n = 3, error bars are s.d., *p < 0.05) (**C**) Relative expression of target genes in MCF7 transiently overexpressing NIK cells cultured with 30 μM FR180204 or DMSO for 24 hrs. (n = 2, error bars are s.e.m) (**D**) Frequency of CSCs in MCF7 control and MCF7 over-expressing cells cultured with 30 uM FR180204 or DMSO during 24 hr. (n = 3, error bars are s.d., *p < 0.04).

The ERK pathway is involved in the activation of many transcription factors, which promote cancer cell growth and tumorigenesis. Interestingly, our results showed that NIK could be regulating the ERK pathway to modulated target genes involved in stemness or epithelial to mesenchymal transition (EMT), a process that gives rise to cancer stem-like cells. To determine the role of NIK in ERK activation, we analyzed the phosphorylation status of ERK in NIK-deficient and NIK-over-expressing cells. Forced expression of NIK promoted ERK phosphorylation, while NIK depletion reduced the expression of phospho-ERK (Fig. 9A). These results suggest that NIK modulates ERK phosphorylation in BCSC.

To further support the role or ERK in the NIK-mediated stem phenotype, we used a chemical inhibitor of this kinase. As expected, ERK inhibition with FR180204 resulted in a reduced expression of the upregulated genes found in the microarray data (Early Growth Response 1, EGR1; Growth Differentiation Factor 15, GDF15; Transcobalamin 1, TCN1 and Dual specificity phosphatase 6, DUSP6) (Fig. 9B).

To verify the role of ERK in the regulation of these target genes, we used specific shRNAs directed against ERK1 and ERK2 (Supplementary Fig. S5A). Our results showed that ERK inhibition also reduced the expression of EGR1 and GDF15, but failed to reduce DUSP6 expression (Supplementary Fig. S5B). Interestingly, NIK-overexpressing MCF7 cells exposed to FR180204 fail to up-regulate target genes (Fig. 9C). Since NIK leads to an increase in ERK activation, we analyzed whether the inhibition of ERK signaling decreases the ability of NIK to enhance CSC fraction. Administration of FR180204 to NIK-overexpressing cells dramatically reduced the CSC fraction to numbers close to those found in MCF7 control cells (Fig. 9D).

## Discussion

It has been proposed that a subpopulation of cancer stem cells are able to initiate and sustain the growth of a tumor^1^, enabling the propagation and formation of metastatic foci at distant sites^26^,^31^,^32^. According to this hypothesis, only CSCs can self-renew indefinitely and differentiate into all tumor cell types^33^. The identification of new CSC targets could be very useful, particularly for neoplasms with high mortality and early relapse rates.

Gene expression profiles have defined the following five breast cancer subtypes: luminal-like, basal-like, HER2 enriched, claudin-low, and normal breast-like^34^,^35^. In breast cancer cell lines, NF-κB activity is differentially represented in luminal and basal breast tumor subtype. Basal breast cancer cell lines possess increased NF-κB activation state and high NIK expression^20^,^21^. NIK expression in breast cancer tissues has not been analyzed; here we show that HER2 breast tumors expressed higher levels of NIK than Triple Negative tumors. Supporting this result, it has been showed that active NF-κB is detected mainly in the HER2+/ER-negative subtype in breast tumor specimens^36^. In addition, it is well known that HER2 overexpression induces NF-κB activation^37^,^38^,^39^. Furthermore, it was reported that HER2 is an important regulator of BCSCs in HER2-positive tumors^40^. HER2 appears to regulate self-renewal in breast tumors lacking HER2 amplification through the receptor activator of NF-κB (RANK)-ligand^41^.

Baldwin AS in 2010 using Her2+/ER- breast cancer cells, showed that HER2 activates NF-kappa B via IKKα and hypothesized that NIK might be activating IKKα since NIK has been associated to the ErbB2 family member EGFR^37^. Nevertheless, the mechanism of how HER2 regulates NIK is not currently known.

In this study, we established a strong association between NIK and the CSC phenotype. We observed that the majority of the stem cell markers analyzed were co-regulated by NIK. Our data showed that NIK expression was higher in BCSCs derived from both luminal and triple negative breast cancer cell lines. NIK depletion reduced the BCSC subpopulation and dramatically impaired malignant characteristics such as tumorigenicity and clonogenicity in both cell lines. Interestingly, even when MCF7 cells expressed low levels of NIK, inhibition of NIK was sufficient to reduce the BCSC population and to impact clonogenic abilities. These results support the strong contribution of this kinase to the stem cell-like phenotype in breast cancer.

Our results showed that NIK expression regulates CSC markers expression, interestingly, ALDH1A1, ALDH1A3 have promoters with putative NF-κB-responsive elements. Analysis of breast cancer tissues revealed that ALDH-expressing cells also express NIK protein in the majority of the tumor analyzed. These results suggest that a NIK-ALDH-dependent regulation of the BCSC population likely operates *in vivo*.

Our data showed that NIK expression negatively regulates CD24. Vesuna *et al.*, found that Twist, an EMT inductor regulated by NF-κB, is able to repress the transcription of CD24^42^. Interestingly, our group has shown that modulation of NIK expression affects Twist levels (data no shown, manuscript in preparation). CD24 has been reported to suppress NF-κB signaling and to potentiate DNA damage-induced apoptosis^43^. Thus, CD24 and NF-κB could be participating in a negative feedback that ultimately determines the final CSC fraction of a tumor.

NIK can activate both canonical and non-canonical NF-κB pathways^44^. NIK preferentially phosphorylates IKKα, although it can also phosphorylate IKKβ in an IKKα-dependent manner^45^. In this article, we demonstrated that NIK overexpression induces NF-κB activation and enhances the expression of p52, p65, and RelB, suggesting that components of non-canonical and canonical NF-κB pathways are involved in NIK-mediated stem cell phenotype modulation. Regulation of NANOG and SOX2 by canonical NF-κB pathway has been described previously^46^, however, this is the first report of regulation of ALDH genes by this pathway.

The role of NF-κB pathways in BCSCs has not been completely elucidated; however, it is clear that NF-κB is involved in BCSCs regulation. In this study, we demonstrated that BCSCs express higher levels of NIK, suggesting that NF-κB performs an important role in BCSCs. Interestingly; NF-κB inhibitors preferentially reduced breast cancer stem-like cell proliferation, with fewer effects on bulk population^47^. Thus, NF-κB disruption could be used as a therapeutic approach to target BCSCs. Still, more work needs to be done to clarify the role of non-canonical and canonical NF-κB pathways in BCSCs. However, there is enough evidence showing that both pathways regulate BCSCs. It is well known that IKKβ-mediated suppression of the canonical NF-κB pathway in the mammary gland significantly reduces CSC populations, impairs mammosphere-forming abilities, and reduces tumorigenicity^46^,^48^. Similarly, IKKα also contributes to BCSC self-renewal, because IKKα^AA/AA^ knock-in mice exhibit delays in the development of breast tumors from cells that cannot develop secondary mammospheres^49^.

In support of our results, a recent study showed that NIK/IKKα promotes breast basal cell expansion and mammosphere-forming ability in a mouse model. The NIK-IKKα cascade acts as an important regulator of the cyclin-dependent kinase inhibitor that enters the nucleus and phosphorylates p27 to stimulate its nuclear export^24^. In addition, it was recently shown that the induction of the non-canonical NF-κB pathway through NIK activation regulates Jagged1 (JAG1) expression and expands the stem cell population in the basal-like breast cancer subtype^21^.

The mechanism by which NIK mediates regulation of the CSC phenotype is poorly understood. Our results showed that NIK regulates the expression of stem cells-related genes through activation of both NF-KB and ERK1/2 pathway. We found that phosphorylation of ERK1/2 is regulated upstream by NIK, and that both NIK and ERK1/2 are required to regulate stemness in breast cancer cells. Supporting our results, Richmond Ann and Dhawan Punita, showed that NIK activates ERK1/2 via MEK1/2 in melanoma cell lines, which have high NIK basal expression. In addition, Gingery in 2008 demonstrated that in osteoclasts, MEK1/2 activates AKT, which in turn phosphorylates NIK^50^,^51^. Among the molecules regulated by ERK1, we found several genes previously described to be highly expressed in various types of CSCs, such as miR-21, EGR1 and GDF15.

Recently, it has been shown that EGR1 plays a critical role in the epithelial-mesenchymal transition (EMT)^52^, a process involved in the acquisition of cancer stem cells properties^53^. EGR1 controls stem cell proliferation^54^, migration and tumor growth^55^,^56^. EGR1 is mainly regulated by the ERK1/2 and NF-κB pathways^57^. Interestingly, EGR1 participates in the EMT by down-regulating E-cadherin through SNAIL^58^. Microarray expression analysis revealed that EGR1 target genes such as GDF15 are also highly expressed in NIK-overexpressing cells. These results suggest that NIK also mediates stem cell phenotype through the induction of genes involved in the EMT process.

Because NIK contributes to the tumorigenic potential of BCSCs, its inhibition could be an effective way to target BCSC and to reduce tumor progression. Due to that NIK is not an essential kinase for canonical NF-κB pathway activation under normal physiological conditions, NIK inhibitors could be safer than more general strategies; however, further studies are required.

## Conclusions

In the present report, we present strong evidence that NIK is a critical kinase for the BCSC phenotype because it modulates stem cell markers, as well as the clonogenic potential and tumorigenicity of these cells. This kinase is an attractive stem cell marker in both luminal and basal tumor types and could possibly be an important therapeutic target.

## Methods

### Cell culture and reagents

MCF7, MDA-MB-231 and SKBR3 cell lines were obtained from the American Type Culture Collection (ATCC, Manassas, VA, USA; www.atcc.org). MCF7 cells were cultured in RPMI 1640 supplemented with 5% fetal bovine serum (FBS), MDA-MB-231 cells were maintained in DMEM supplemented with 5% FBS and SKBR3 were cultured in McCoy’s 5A supplemented with 10% FBS. All cell lines were cultured in a humidified atmosphere at 37 °C with 5% CO_2_.

### Flow cytometry analysis

Cells were detached with Accutase (Life Technologies, Carlsbad, CA, USA) and washed with Phosphate buffered saline (PBS) 1% and resuspended in the stain buffer (PBS with FBS 1%). A total of 1 × 10^6^ cells were incubated with the FITC-CD44 (555478, BD Biosciences) and PE-CD24 (555428, BD Biosciences) for 30 min on ice. MDA-MB-231 cells were also labeled with APC-ESA (347200, BD Biosciences) for 30 min. FITC-IgG1(130-092-213, Miltenyi Biotec), PE-IgG1 (130-092-212, Miltenyi Biotec) and APC-IgG1 (130-092-214, Miltenyi Biotec) antibody isotype were used as controls. For cell sorting, 1 × 10^8^ MCF7 cells were sorted into CD44^+^/CD24^−/low^ and CD44^+^/CD24^+^ subsets. MDA-MB-231 cells were sorted into ESA^+^ and ESA^−^ subsets. Cells were sorted on a FACSAria (fluorescence-activated cell sorter, Becton Dickinson, Franklin Lakes, NJ, USA) and then were harvested and grown under standard culture conditions for 24 h prior to any further procedures.

ALDH+ population was separated by FACS. ALDH1 activity was determined using the Aldeflour assay (Stem Cell Technologies) according to the manufacturer’s instructions. Briefly, 1 × 10^6^ cells were suspended in Aldeflour assay and incubated with 5 ul of activated Aldeflour reagent during 30 minutes. Control cells were incubated with Aldeflour reagent along with 5 ul of DEAB solution. The brightly fluorescent ALDH1-expressing cells were detected in the FITC channel on a FACSAria cell sorter. Cells were harvested and grown under standard culture condition for 24 h prior to RNA isolation.

### RT-PCR and quantitative real-time PCR

Total RNA was isolated with TRIzol reagent (Life Technologies) according to the manufacturer’s instructions. Then, 2 μg of RNA was reverse transcribed with a Maxima First strand cDNA synthesis kit (Thermo Fisher Scientific, Rockford, IL, USA). RT-qPCR was performed by the 7900HT (Applied Biosystems) or the QuantStudio 7. PCR was performed with the SYBR-select Master Kit (Applied Biosystems). Primers listed in Supplementary Table S4 were used for qRT-PCR.

### Protein preparation and Western Blotting

Total proteins were extracted with RIPA buffer (Upstate Biotechnology, Inc., Lake Placid, New York, USA) supplied with protease and phosphatase inhibitors. Subcellular fraction was carried out with the Subcellular Protein Fractionation Kit (Thermo Scientific, MA, USA) according to the manufacturer’s instructions. Proteins were resolved in 10% SDS-PAGE and transferred onto polyvinylidene difluoride (PVDF) membranes (Millipore, Bedford, MA, USA). Membranes were blocked with 5% non-fat dry milk in Tris-buffered saline with 0.1% Tween (TBST) for 2 h, then were incubated overnight with the corresponding antibodies at 4 °C, followed by incubation with horseradish peroxidase-conjugated goat anti-mouse IgG (W402B, Promega, Madison, WI, USA) or goat anti-rabbit IgG (W401B, Promega), or rabbit anti-Goat IgG (Zymax, Life Technologies, Carlsbad, CA, USA) as appropriate, for 1 h at RT in TBST. Antibody binding was detected with the Immobilon Western kit (Millipore), and images were visualized with the VersaDoc Imaging System (BioRad, Hercules, CA, USA). The antibodies used were, anti-p65 (1:1000, sc372, Santa Cruz Biotechnology CA, USA), anti-RelB (1:1000, sc28689, Santa Cruz Biotechnology), anti-p50 (1:1000, sc1190, Santa Cruz Biotechnology), anti-p52 (1:1000, 05–361, Millipore), anti-p-ERK1/2 (1:10000, Cell Signaling), anti-αtubulin (1:2000, sc53646, Santa Cruz Biotechnology), anti-GAPDH (1:5000, sc25778, Santa Cruz) and anti-Laminin A/C (1:1000, 4777, Cell Signaling).

### shRNA experiments

To decrease NIK expression, two shRNAs (1: CCGAGAGTCTTGATCAGAT, 2: GGTCAACATCTTCATGGA, and 3: GAGGAATACCTAGTGCAT,) were designed with the RNAi Target Sequence Selector (Clontech). To decrease ERK1 and ERK2 expression, we designed two shRNAs for each gene (1: GGATCAGCTCAACCACATT, 2: CCTCCAACCTGCTCATCAA, 3: CACCAACCATCGAGCAAAT, 4: CAGCCAGGATACAGATCTT). All shRNAs were cloned into the pSIREN RQ-vector (Clontech). shRNA against luciferase was used as a control. MCF7, MDA-MB-231 and SKBR3 cells were transfected and then selected with 0.5-μg/ml puromycin (Sigma-Aldrich, St. Louis, MO, USA), 7.5 μg/ml puromycin or 0.6 μg/ml for 3 weeks respectively.

### ERK pathway inhibition

A selective ERK inhibitor FR180204 (Tocris, Biosciences) was employed in MCF7 cells to explore the role of ER in stemness. Briefly MCF7 control or overexpressing NIK cells were treated with 30 μM for 24 h. As a control, cells were treated with an equivalent volume of DMSO. ERK inhibition was verified in a western blot assay.

### NIK overexpression experiments

To overexpress NIK, we generated an expression vector by recombining a pENTR221 vector (Life Technologies) containing the ORF of NIK with the pTREX-DEST30 vector (Life Technologies). MCF7 and SKBR3 cells were transfected with 4 μg of (pT-Rex-NIK), in the presence of LTX-Lipofectamine for 24 h. Stable MCF7 and SKBR3 clones were selected with 300 μg/ml or 700 μg/ml of G418 (GIBCO) respectively for 3 weeks. As a control, MCF7 and SKBR3 cells were transfected with 4-μg pTREX-Lacz.

### NF-κB reporter assays

NF-κB reporter assays were performed by generating a stable MCF7 cell line bearing an NF-κB reporter vector. These cells were co-transfected with 4 μg of pT-Rex-NIK, pT-Rex-Lac, shNIK1, shNIK2 and 1 μg of pCMV-Sport-Gal (Life Technologies) using LTX-Lipofectamine. The enzymatic activities of both the firefly luciferase and the beta-galactosidase reporter genes were determined 48 h after transfection with a Dual-luciferase Reporter Assay kit (Promega) and a Luminescent Beta-galactosidase Detection Kit (Clontech), respectively.

### Immunofluorescence

Immunofluorescence analysis was performed with the following primary antibodies: p52 (1:500, sc848, Santa Cruz); p65 (1:500, sc372, Santa Cruz Biotechnologies); RelB (1:500, sc28689, Santa Cruz Biotechnology); P50 (1:250, sc1190, Santa Cruz Biotechnologies), NIK (1:1000, 4994, Cell Signaling), phospho-p52 (1:500, Cell Signaling), phosphor-p65 (1:500, 3036, Cell Signaling). Briefly, 5 × 10^5^ cells were washed with PBS, fixed in 4% paraformaldehyde (Sigma-Aldrich, St. Louis, MO, USA) for 20 min, and permeabilized in 0.1% of Triton 100× (Sigma-Aldrich) for 30 min. Cells were washed and blocked with 5% Bovine serum albumin (BSA) for 1 h and subsequently incubated with primary antibodies for 2 h at RT, followed by incubation with the secondary Cy3-goat anti-mouse antibody (Millipore) or Cy3-goat anti-rabbit antibody (Millipore) or Cy3-rabbit-anti-goat (811615, Zymed) for 1 h. Cells were then washed and slides were mounted in Everbrite mounting medium with 6-Diamidine-2′-phenylindole dihydrochloride (DAPI, Biotium, Inc., Hayward, CA, USA). Fluorescence analysis was performed on a confocal microscope (Zeiss LSM 510).

### *In vivo* tumorigenic assays

Several cell dilutions of MCF7, MDA-MB-231 or SKBR3 cells were suspended in 100 ml of PBS with 50% growth factor-reduced Matrigel (BD Biosciences, Bedford, MA, USA) and were subcutaneously injected into *nu/nu* female mice aged 4–6 weeks. Female athymic Balb-c nu/nu mice, between 6–8 weeks, were supplied by the Autonomous Metropolitan University, Mexico City, México. The animals were kept in a pathogen-free environmental and fed ad libitum. All animal procedures reported in the present paper were performed according to the NIH Animal Use and Care Guidelines (USA). The local institutional committee: Comite Interno de Cuidados de Animales of the Instituto Nacional de Cancerologia (CICUAL-INCan) approved the procedures according to all applicable National institutional and governmental regulations concerning the ethical use of experimental animals. MCF7 transplants were allowed to grow for 150 days; SKBR3 and MDA-MB-231 were allowed to grow for 60 days. After this interval or when the tumors reached a maximum volume of 700 mm^3^, animals were sacrificed by cervical dislocation trying to minimize suffering.

#### Viral cell transduction and bioluminescence imaging

MDA-MB-231 shLuc or shNIK cells expressing Luciferase2-eGFP were generated as described previously^59^. Stable cells were selected with 200 μg/ml of Zeocin (life technologies) for 4 weeks. Mice were given an intraperitoneal (i.p.) injection with 200 μl of D-luciferin (30 mg/mL). Mice were anesthetized with isoflurane (2% in 1 L/min oxygen), and bioluminescence images were acquired 10–15 min after D-luciferin injection using the IVIS XR system (Caliper Life Sciences, Hopkinton MA).

### Colony-forming assays

Briefly, 1,000 or 4000 MCF7 single cells were seeded in DMEM medium containing 0.3% low-melting-point agarose and 5% FBS, and the cells were plated on 12 or 6-well plates coated with 0.5% low-melting-point agarose. After 40 days in culture, colonies were stained with 0.1% crystal violet and counted using ImageJ software. For clonogenic assays of the MDA-MB-231 cell line, cells were seeded in 96 well plates as described in results. After two weeks in culture, cells were stained with 0.1% crystal violet.

### Sphere formation assays

Briefly, 2.5 × 10^4^/ml single cells were seeded over non-adherent suspension culture flasks. Cells were cultured in Mammocult (StemCell technologies) supplemented with 0.48 μg/ml hydrocortisone and 4 μg/ml heparin. After 7 days, the spheres were counted and were disaggregated with trypsin until singles cells were obtained. Single cells were again seeded and spheres were counted after seven days. The same number of cells was seeded for each cell line.

### Tissue microarray construction

Tumor tissues from 191 patients were embedded in paraffin, and 4-mm sections were stained with H&E to select morphologically representative areas. Tissues selected were punched with a 1-mm needle and transferred onto a recipient paraffin block using a Tissue Microarray ATA 100 Chemicon (Chemicon, Temecula, CA, USA). Then, 4-mm sections of these tissue arrays were sectioned and placed onto positively charged slides (VWR Superfrost Plus).

### Immunohistochemical analysis of breast cancer tissues

Tissue sections were stained with Estrogen Receptor (SP1, CONFIRM), Progesterone Receptor (IE2, CONFIRM), and HER2 (4B5, PATHWAY). Immunohistochemistry was performed using the UltraView Universal DAB Detection Kit (Ventana). The immunohistochemistry of NIK (1:500, ab220442) and ALDH (1:500, 611195, BD) was performed using a Ventana Ultraview DAB detection kit (Ventana) and UltraView Universal Alkaline Phosphatase Red Detection Kit (Ventana), respectively. Diaminobenzidine or Fast Red was employed as the chromogen, and hematoxylin as the nuclear counterstain. All slides were processed in a Ventana BenchMark XT processor (Ventana, Tucson, AZ, USA). Slides were scanned in ScanScope CS2 (Aperio) and image analysis was conducted in ImageJ. This study has used paraffin embedded breast cancer tissue obtained from the Oncology Hospital in the XXI Century National Medical Center. All used tissues were part of residual material and they did not endanger patients diagnostic.

### Genome-Wide Microarray analysis

Total RNA from MCF7 cells transfected with shNIK, shLUC or pt-REX-DEST-NIK was obtained 48 hrs after transfection. All samples were treated with DNAse 1 (Ambion). RNA integrity was evaluated with a Bioanalyzer 2100 (Agilent) and only samples with an RNA Integrity Number (RIN) greater than 9 were used. Three independent biological replicates were used and hybridized into Affymetrix Gene ST 2.0 Arrays at the INMEGEN microarray core unit. Data were deposited in the Gene Expression Omnibus (GEO submission GSE63382)

### Statistical analysis

Flow cytometry data were analyzed with the Flowjo software package (Treestar, Ashland, OR, USA). Densitometric analysis was performed using ImageJ software. Statistical analysis was carried out using GraphPad, and a 5% level was considered significant. Analysis of NIK and ALDH expression in breast cancer patients were conducted using JMP and One-way Analysis of variance (ANOVA) was employed to determine significant differences. Microarray data analysis was performed using bioconductor packages. Data were normalized with the Robust Multi-array Average method (RMA) contained in the oligo package.

The Limma R package was used to detect differentially expressed^60^. Genes with a >1.8 fold change and a crude p < 0.05 were considered for further analysis. Ingenuity Pathway Analysis (Qiagen, USA) software was used to identify enriched networks.

## Additional Information

**How to cite this article**: Vazquez-Santillan, K. *et al.* NF-kappaB-inducing kinase regulates stem cell phenotype in breast cancer. *Sci. Rep.* **6**, 37340; doi: 10.1038/srep37340 (2016).

**Publisher’s note:** Springer Nature remains neutral with regard to jurisdictional claims in published maps and institutional affiliations.

## Supplementary Material

Supplementary Information
