# Corrigendum: NF-kappaB-inducing kinase regulates stem cell phenotype in breast cancer

**DOI:** 10.1038/srep44971

**Published:** 2017-03-23

**Authors:** Karla Vazquez-Santillan, Jorge Melendez-Zajgla, Luis Enrique Jimenez-Hernandez, Javier Gaytan-Cervantes, Laura Muñoz-Galindo, Patricia Piña-Sanchez, Gustavo Martinez-Ruiz, Javier Torres, Patricia Garcia-Lopez, Carolina Gonzalez-Torres, Victor Ruiz, Federico Avila-Moreno, Marco Velasco-Velazquez, Mayra Perez-Tapia, Vilma Maldonado

Scientific Reports 6: Article number: 3734010.1038/srep37340; published online: 11 23 2016; updated: 03 23 2017

The Acknowledgements section in this Article is incomplete.

“Karla Itzel Vazquez Santillan was supported by a fellowship from CONACYT (165306), and this study is part of her doctoral thesis from the Biomedical Sciences Doctorate Program, Faculty of Medicine, Universidad Nacional Autonoma de Mexico. This project was partially supported by CONACYT grant CB-2009-01-132931”.

should read:

“Karla Itzel Vazquez Santillan is a doctoral student from Programa de Doctorado en Ciencias Biomédicas, Universidad Nacional Autónoma de México (UNAM) and received fellowship 165306 from CONACYT. This project was partially supported by CONACYT grant CB-2009-01-132931”.

