# Supplementary material for: NF-kappaΒ-inducing kinase regulates stem cell phenotype in breast cancer
Source: Sci Rep. 2016 Nov 23;6:37340. doi: 10.1038/srep37340 (PMC5120353; doi:10.1038/srep37340)
Supplement: Supplementary Information [file srep37340-s1.pdf]

Supplementary information.

NF-kappaB-inducing kinase regulates stem cell phenotype in breast cancer.

Karla Vazquez-Santillan[1], Jorge Melendez-Zajgla[1], Luis Enrique Jimenez-Hernandez,[1] Javier Gaytan-Cervantes[1], Laura Muñoz-Galindo[1], Patricia Piña-Sanchez[2], Gustavo Martinez-Ruiz[1], Javier Torres[3], Patricia Garcia-Lopez[4], Carolina Gonzalez-Torres[1], Victor Ruiz[5], Federico Avila-Moreno[6] Marco Velasco-Velazquez[7], Mayra Perez-Tapia[8], Vilma Maldonado[1,].*

[1]Instituto Nacional de Medicina Genómica (INMEGEN), México, 14610, México.

[2]Unidad de Investigación Médica en Enfermedades Oncológicas (UIMEO), Hospital de Oncología IMSS, México.

[3]Unidad de Investigación Médica en Enfermedades Infecciosas y Parasitarias (UMAE), Hospital de Pediatría, IMSS, México.

[4]Instituto Nacional de Cancerología (INCAN)

[5]Instituto Nacional de Enfermedades Respiratorias "Ismael Cosío Villegas" (INER), México.

[6]Facultad de Estudios Superiores Iztacala, UNAM, México.

[7]Facultad de Medicina UNAM, México.

[8]Unidad de Desarrollo e Investigación en Bioprocesos (UDIBI) y Departamento de Inmunología, IPN

* Corresponding author: Vilma Maldonado vilmaml@gmail.com

Karla Vázquez Santillán: kivazquez@inmegen.gob.mx

Jorge Melendez Zajgla: jmelendez@inmegen.gob.mx

Luis Enrique Jiménez Hernández: ljimenez_035@gmail.com

Patricia Piña Sanchez: patricia_1307@yahoo.com.mx

Javier Gaytan Cervantes: alternativesplicing@gmail.com

Laura Muñoz Galindo: lauramunozg@gmail.com

Gustavo Martinez Ruiz: gustavobambam@gmail.com

Javier Torres: jtorresl57@yahoo.com.mx

Patricia Garcia Lopez: pgarcia_lopez@yahoo.com.mx

Carolina Gonzalez Torres: gonzaleztorrescaro@gmail.com

Victor Ruiz: vicoruz@yahoo.com.mx

Federico Avila Moreno: f.avila@campus.iztacala.unam.mx

Marco Velasco Velázquez: marcovelasco@hotmail.com

Mayra Pérez Tapia: maypet@hotmail.com

Supplementary Table 1. Tumor formation of BCSCs and non-BCSCs of in immunodeficient *nu/nu* mice.

Subpopulation	Cell number	Tumors formed
MCF7 CD44+/CD24-	1×10^5	3/3
MCF7 CD44-/CD24+	1×10^5	0/3
MDA-MB-231 EpCAM+	1×10^5	3/3
MDA-MB-231 EpCAM-	1×10^5	1/3

Supplementary Table 2. Genes regulated in MCF7 overexpressing-NIK cells

Symbol	Definition	Fold Change	raw p-value
ACRC	acidic repeat containing	-2.997798332	0.000253853
TSNAXIP1	translin-associated factor X interacting protein 1	-2.938635998	0.008106129
PDE5A	phosphodiesterase 5A, cGMP-specific	-2.763263867	0.025233844
FOXP2	forkhead box P2	-2.70920582	0.002484034
MUC4	mucin 4, cell surface associated	-2.592534762	0.005874116
SCG3	secretogranin III	-2.509534883	0.021167533
EFCAB5	EF-hand calcium binding domain 5	-2.324257643	0.000124181
C5orf60	chromosome 5 open reading frame 60	-2.298995008	0.019017708
CYP4F25P	cytochrome P450, family 4, subfamily F, polypeptide 25, pseudogene	-2.275026473	0.002286685
CCRL1	chemokine (C-C motif) receptor-like 1	-2.270093416	0.004832252
INS	insulin	-2.190474369	0.016429673
ABCB1	ATP-binding cassette, sub-family B (MDR/TAP), member 1	-2.187303375	0.005396647
ADORA3	adenosine A3 receptor	-2.170413611	0.039321949
IGLV2-23	immunoglobulin lambda variable	-2.113254712	0.008531093
GRM6	glutamate receptor, metabotropic 6	-2.111819699	0.026817403
CDH18	cadherin 18, type 2	-2.082678284	0.007945737
GBGT1	globoside alpha-1,3-N-acetylgalactosaminyltransferase 1	-2.048682856	0.000232321
ITGA8	integrin, alpha 8	-2.044686982	0.00810342
PRH2	proline-rich protein HaeIII subfamily 2	-2.027146001	0.009538984
CYP19A1	cytochrome P450, family 19, subfamily A, polypeptide 1	-2.00904981	0.016221972
GPM6B	glycoprotein M6B	-2.00784792	0.03724353
KIAA1045	KIAA1045	-1.976146632	0.007334539
RN7SL472P	RNA, 7SL, cytoplasmic 472, pseudogene	-1.973108102	0.00014591
LEPREL1	leprecan-like 1	-1.964104468	0.025005985
TPD52L3	tumor protein D52-like 3	-1.952401818	0.012042181
GLB1L3	galactosidase, beta 1-like 3	-1.926179592	0.004089145
SERPINB3	serpin peptidase inhibitor, clade B (ovalbumin), member 3	-1.924099403	0.051862256
NEFL	neurofilament, light polypeptide	-1.904413375	0.000149016
ALK	anaplastic lymphoma receptor tyrosine kinase	-1.890074804	0.03084883
TEX12	testis expressed 12	-1.885481942	0.003118034
CCL26	chemokine (C-C motif) ligand 26	-1.86276197	0.03401562
PTPRN	protein tyrosine phosphatase, receptor type, N	-1.853929864	0.050321571
PLCL2	phospholipase C-like 2	-1.834573874	0.009271919
snoU13	Small nucleolar RNA U13	-1.83028714	7.96E-05
SULT1C3	sulfotransferase family, cytosolic, 1C, member 3	-1.829610647	0.000149746
CATSPERB	catsper channel auxiliary subunit beta	-1.808865326	0.017453792
PDE6B	phosphodiesterase 6B, cGMP-specific, rod, beta	-1.806169556	0.023858459
GDF15	growth differentiation factor 15	1.523377332	0.000567534
BIRC3	baculoviral IAP repeat containing 3	1.50941989	0.003332765

DEPDC4	DEP domain containing 4	1.800686047	0.002331078
ZNF595	zinc finger protein 595	1.805241271	0.000168266
AICDA	activation-induced cytidine deaminase	1.834843056	0.002187369
ENKUR	enkurin, TRPC channel interacting protein	1.847396235	0.014241283
UGT1A6	UDP glucuronosyltransferase 1 family, polypeptide A6	1.856561792	0.026398801
BTBD8	BTB (POZ) domain containing 8	1.862180174	0.011850701
TLDC1	TBC/LysM-associated domain containing 1	1.864019131	0.008675778
PYHIN1	pyrin and HIN domain family, member 1	1.880709035	0.03904283
FAM98B	family with sequence similarity 98, member B	1.90663894	0.019620982
U6	U6 spliceosomal RNA	1.90878772	0.008523232
CEP170P1	centrosomal protein 170kDa pseudogene 1	1.924512889	0.001725804
MAP3K14-AS1	MAP3K14 antisense RNA 1	1.944119185	5.66E-05
ATP8B5P	ATPase, class I, type 8B, member 5, pseudogene	1.94815257	0.015025654
MRPL30	mitochondrial ribosomal protein L30	1.953222993	0.011717307
CTSE	cathepsin E	1.958464983	0.005720327
Y_RNA	Y RNA	1.959383776	0.000963941
SKAP1	src kinase associated phosphoprotein	1.965296066	0.004595609
SH3BP2	SH3-domain binding protein 2	1.978252618	0.00273059
ELMOD3	ELMO/CED-12 domain containing 3	1.989620577	0.002571864
ZKSCAN3	zinc finger with KRAB and SCAN domains 3	1.991713312	0.006112225
ADPRM	ADP-ribose/CDP-alcohol diphosphatase, manganese-dependent	1.993306183	0.009358243
NCF1	neutrophil cytosolic factor 1	1.99735393	0.002575318
ANKRD18B	ankyrin repeat domain 18B	2.000429797	0.010149451
MIR21	microRNA 21	2.010043421	0.001670391
EML2	echinoderm microtubule associated protein like 2	2.051818806	0.037724416
TTC18	tetratricopeptide repeat domain 18	2.11207344	0.00159627
SLC12A6	solute carrier family 12 (potassium/chloride transporters), member 6	2.144839955	0.011965818
DUSP6	dual specificity phosphatase 6	2.156315579	0.000486362
IFNA7	interferon, alpha 7	2.185242419	0.014898222
SSBP1	single-stranded DNA binding protein 1, mitochondrial	2.197642425	0.007923309
SSUH2	ssu-2 homolog (C. elegans)	2.243788458	0.005073442
CCDC158	coiled-coil domain containing 158	2.271969289	0.017940798
TCN1	transcobalamin I (vitamin B12 binding protein, R binder family)	2.294436691	0.000296253
IGLV2-11	immunoglobulin lambda variable 2-11	2.350845945	0.007979251
CGRRF1	cell growth regulator with ring finger domain 1	2.423042987	0.036436367
TCP11L1	t-complex 11, testis-specific-like 1	2.448389919	3.08E-05
COL5A2	collagen, type V, alpha 2	2.499783814	0.001452101
EGR1	early growth response 1	2.527017812	1.72E-05
TRAJ17	T cell receptor alpha joining 17	2.550658935	0.014304261
CCPG1	cell cycle progression 1	2.874783545	0.048506819
MAP3K14	mitogen-activated protein kinase kinase kinase 14	10.76089871	2.27E-07

Supplementary Table 3.- Genes regulated in deficient NIK cells

Symbol	Definition	Fold Change	P-value
PRKG1	protein kinase, cGMP-dependent, type I	-2.520092461	1.55E-05
OC90	Otoconin-90	-2.487747391	0.002698203
IGLV4-3	immunoglobulin lambda variable 4-3	-2.444794721	0.000335441
ALLC	allantoicase	-2.258502744	0.005631608
NEFL	neurofilament, light polypeptide	-2.248994293	0.006125414
CSPG4	chondroitin sulfate proteoglycan 4	-2.220477573	0.008242372
MAP2K6	mitogen-activated protein kinase kinase 6	-2.148614509	0.00686966
DENND3	DENN/MADD domain containing 3	-2.102122387	0.014025744
LEPREL1	leprecan-like 1	-2.067513381	0.005735957
CPLX1	complexin 1	-2.001511632	0.015089237
GLB1L3	galactosidase, beta 1-like 3	-1.986285953	0.000152846
NXF3	nuclear RNA export factor 3	-1.931346028	0.002604077
TEX12	testis expressed 12	-1.914516375	0.000801005
CCDC150	coiled-coil domain containing 150	-1.902755249	0.015637514
CCRL1	chemokine (C-C motif) receptor-like 1	-1.881517446	0.000412245
C6	complement component 6	-1.877027777	0.001394614
TAL1	T-cell acute lymphocytic leukemia 1	-1.876030565	0.000512159
TPD52L3	tumor protein D52-like 3	-1.873855882	0.010192919
CHRNA7	cholinergic receptor, nicotinic, alpha 7 (neuronal)	-1.844338332	0.000130025
AC013269.5	Uncharacterized protein	-1.827686126	0.006476783
AC140481.2	Uncharacterized protein	-1.827686126	0.006476783
RYBP	RING1 and YY1 binding protein	1.825957686	0.000483636
SUMF1	sulfatase modifying factor 1	1.829762837	0.005893955
SSBP1	single-stranded DNA binding protein 1, mitochondrial	1.83046899	0.007488112
OLFM3	olfactomedin 3	1.863717679	0.014594876
MT-TT	mitochondrially encoded tRNA threonine	1.87272189	0.000123071
TCP11L1	t-complex 11, testis-specific-like 1	1.881287057	0.002615535
U6	U6 spliceosomal RNA	1.885316407	0.001065489
PRG4	proteoglycan 4	1.913008565	0.003799423
DNAH14	dynein, axonemal, heavy chain 14	1.913888345	0.005696986
U6	U6 spliceosomal RNA	1.923352684	0.000749655
SLC4A5	solute carrier family 4, sodium bicarbonate cotransporter, member 5	1.945727939	0.001113093
CCDC129	coiled-coil domain containing 129	1.946438372	0.004677811
WDR19	WD repeat domain 19	1.953362898	0.002967937
SSUH2	ssu-2 homolog	1.978769179	0.014936233
U6	U6 spliceosomal RNA	1.996338917	0.012040764
RNY1P5	RNA, Ro-associated Y1 pseudogene 5	2.006183169	0.012311836
TRDN	triadin	2.006973638	0.001042032

CDC14B	cell division cycle 14B	2.041370902	0.000104669
CYP2D6	cytochrome P450, family 2, subfamily D, polypeptide 6	2.047996619	0.00296561
UBAC2	UBA domain containing 2	2.052122233	0.000566799
BTBD9	BTB (POZ) domain containing 9	2.065412582	0.003121109
IL7	interleukin 7	2.069506338	1.12E-05
ANGPTL1	angiopoietin-like 1	2.094167249	0.012606112
ACTR3B	ARP3 actin-related protein 3 homolog B (yeast)	2.135839641	0.000273097
ATP8B5P	ATPase, class I, type 8B, member 5, pseudogene	2.14827696	0.001855622
SOCS6	suppressor of cytokine signaling 6	2.212969141	0.003464457
GBA3	glucosidase, beta, acid 3 (cytosolic)	2.259363919	0.012010093
NDUFA5	NADH dehydrogenase (ubiquinone) 1 alpha subcomplex, 5, 13kDa	2.438972233	0.005192536
STON1-GTF2A1L	STON1-GTF2A1L readthrough	2.441644791	7.44E-05
GNAI1	guanine nucleotide binding protein (G protein), alpha inhibiting activity polypeptide 1	2.567207661	0.000516532
BOC	BOC cell adhesion associated, oncogene regulated	2.707872854	0.000342732
TRAV1-2	T cell receptor alpha variable 1-2	2.830257532	0.005508257

Supplementary Table 4.- Primers used for q-RT-PCR analysis.

Name	Primer	Sequence	TM
ALDH1A1	Forward	TGTTAGCTGATGCCGACTTG	56
	Reverse	TTCTTAGCCCGCTCAACACT	
ALDH1A3	Forward	GCATACCGTGGAAGGGCG	57
	Reverse	GCTCTCTGGGCTATTGATTCTGTC	
ALDH8A1	Forward	CAGGCTACTTTATGCTTCCCAC	55
	Reverse	GCTCTTTCAATCACCTCCTCTT	
OCT4	Forward	ACATCAAAGCTCTGCAGAAAGAAC	56
	Reverse	CTGAATACCTTCCCAAATAGAACCC	
SOX2	Forward	AGGAGTTGTCAAGGCAGAG	56
	Reverse	CCGCCGATGATTGTTATT	
NANOG	Forward	AGGCAAACAACCCACTTCTG	57
	Reverse	TCTGCTGGAGGCTGAGGTAT	
NIK	Forward	TTAGAAACACCCCGCAGTTC	56
	Reverse	TCCACACGTGGTTCAGACAT	
TBP	Forward	TGCCACACCCTGCAACTCAACA	60
	Reverse	CCCCTTCATTGACCTCAACT	
EGR1	Forward	AGAGCCGCACCCAGCAGCCT	60
	Reverse	GGGCAAGCGTAAGGGCGTTCGT	
DUSP6	Forward	GTGACAACAGGGTTCCAGCACAGCA	60
	Reverse	GGCCAGACACATTCCAGCAAGGAGG	
TCN1	Forward	TGGGAACTACTCAAC CGCCGA	60
	Reverse	GGTCAGAGCCAGGACAGCCA	
MAP2K6	Forward	TCTGACATTTGGAGTCTGGGCATCA	60
	Reverse	GTCTGCTGGGAGTTGTGGCG	
GDF15	Forward	TGAGACACCCGATTCCTGCCCAAA	60
	Reverse	CAGCCCCCGAGTCCCCAAGA	
EGR1	Forward	AGAGCCGCACCCAGCAGCCT	60
	Reverse	GGGCAAGCGTAAGGGCGTTCGT	
CSPG4	Forward	GCCTTCACCAACTTCTCCTCCTCT	60
	Reverse	TGACGTTCACTACGGCTGATGC	
ERK2	Forward	CTGTTCCCAAATGCTGACTCCAAA	60
	Reverse	GCCAGAGCCTGTTCTACTTCAATC	
ERK1	Forward	CATTCTGGGCATCCTGGGCTC	60
	Reverse	GGAGGGCAGAGACTGTAGGTAGTTT	

Supplementary Table 5. Expression of NF-κB inducing kinase (NIK) in breast cancer patients by immunohistochemistry.

Percentage of cells	NIK+ patients	ALDH+ patients
0 %	39 (20.5%)	85 (44.7%)
1-10 %	30 (15.7%)	55 (28.9%)
11-50 %	47 (24.7%)	29 (15.2%)
51-70 %	17 (8.9%)	11 (5.9%)
70-100 %	57 (30%)	10 (5.2%)
Total cases	190	190

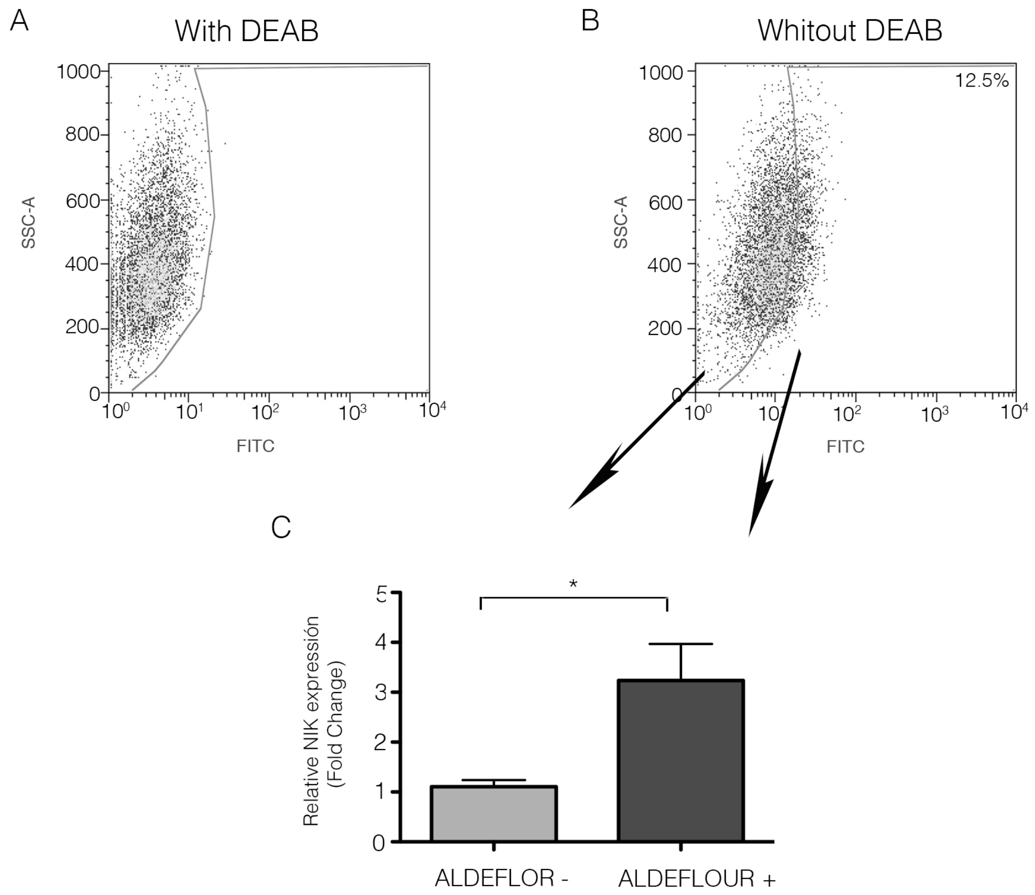

SUPPLEMENTARY FIGURE 1. NIK is enriched in MDA-MB-231 cells exhibiting ALDH activity. (A-B): ALDH activity was evaluated using the ALDEFLOUR assay kit. A total of $1x10^6$ MDA-MB-231 cells were incubated with 5ul of activated ALDEFLOUR substrate during 30 min. (A): DEAB was used as a control to establish the baseline fluorescence of these cells. (B): Incubation of cells without DEAB induces a shift in fluorescence defining the population with ALDH activity. (C): NIK expression was analyzed by Real Time PCR in ALDEFLOUR negative and positive cells. Data are expressed as fold change relative to ALDEFLOUR negative cells (n=2, error bars are +/- s.e.m)

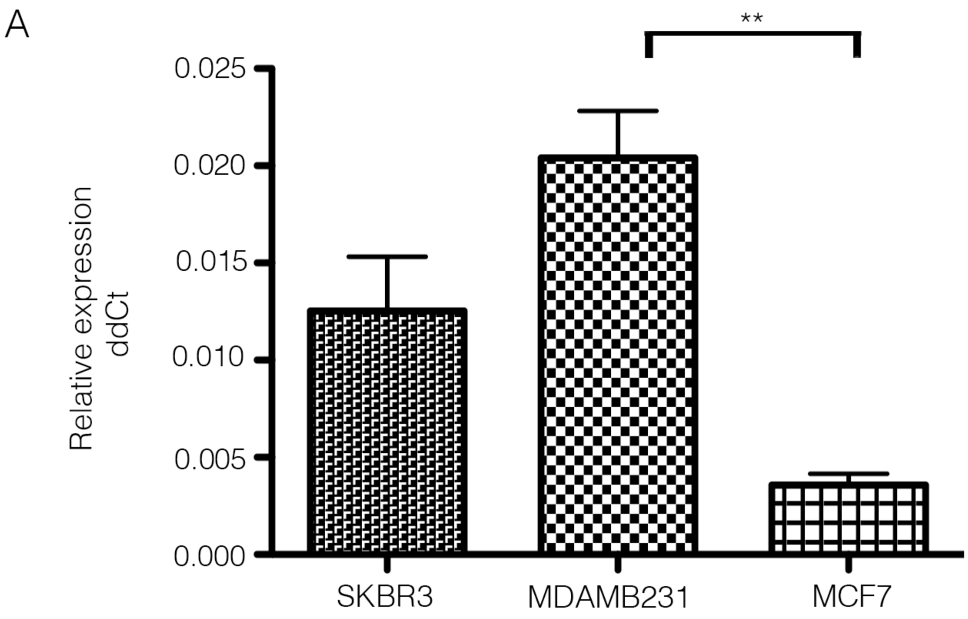

SUPPLEMENTARY FIGURE 2. NIK expression in breast cancer cell lines. NIK expression was analyzed by Real Time PCR in three breast cancer cell lines. Data are expressed as relative expression (n=3, error bars are +/- s.e.m, **p=0.005)

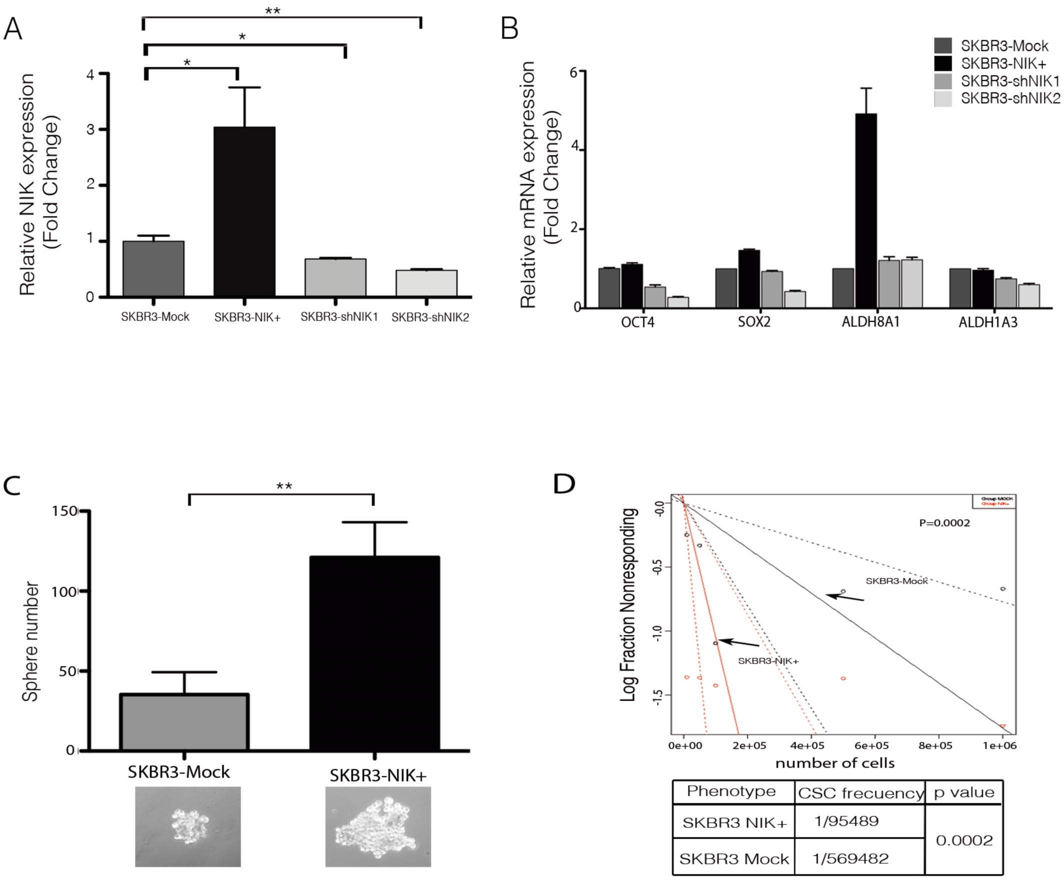

Phenotype	CSC frecuency	p value
SKBR3 NIK+	1/95489	0.0002
SKBR3 Mock	1/569482	

SUPPLEMENTARY FIGURE 3. NIK regulates stemness in SKBR3 cells. (A) To modulate NIK expression, two shRNA and an expression vector were used in SKBR3 cells. NIK expression was analyzed by Real Time PCR and normalized to TBP. Data are expressed as fold change relative to Mock cell (n=3, error bars a *P<0.05). (B): The level of CSC markers was analyzed in stable NIK-overexpressing and in NIK depleted SKBR cells. Data are expressed as fold change relative to Mock cell (n=3, error bars a *P<0.05). (C): Quantification of mammosphere number derived from SKBR mock cells (SKBR3-Mock) and NIK-overexpressing SKBR3 cells (SKBR3-NIK+) in both first and second passages. For mammosphere assay, 2.5 x 10^5 cells/ml of each cell line were seeded. (n=3, error bars are s.e.m., *p< 0.04). (H): A total of 16 mice were inoculated with 10000 (n=4), 50000 (n=4), 100000 (n=4), 500000 (n=4) and 1000000 (n=4) NIK-overexpressing and SKBR3 control cells. SKBR3 stable NIK-overexpressing cells were inoculated into the right flanks and SKBR3 control cells in the left flank. Tumors were monitored each week during 45 days. The frequency of cancer stem cells was calculated using ELDA software.

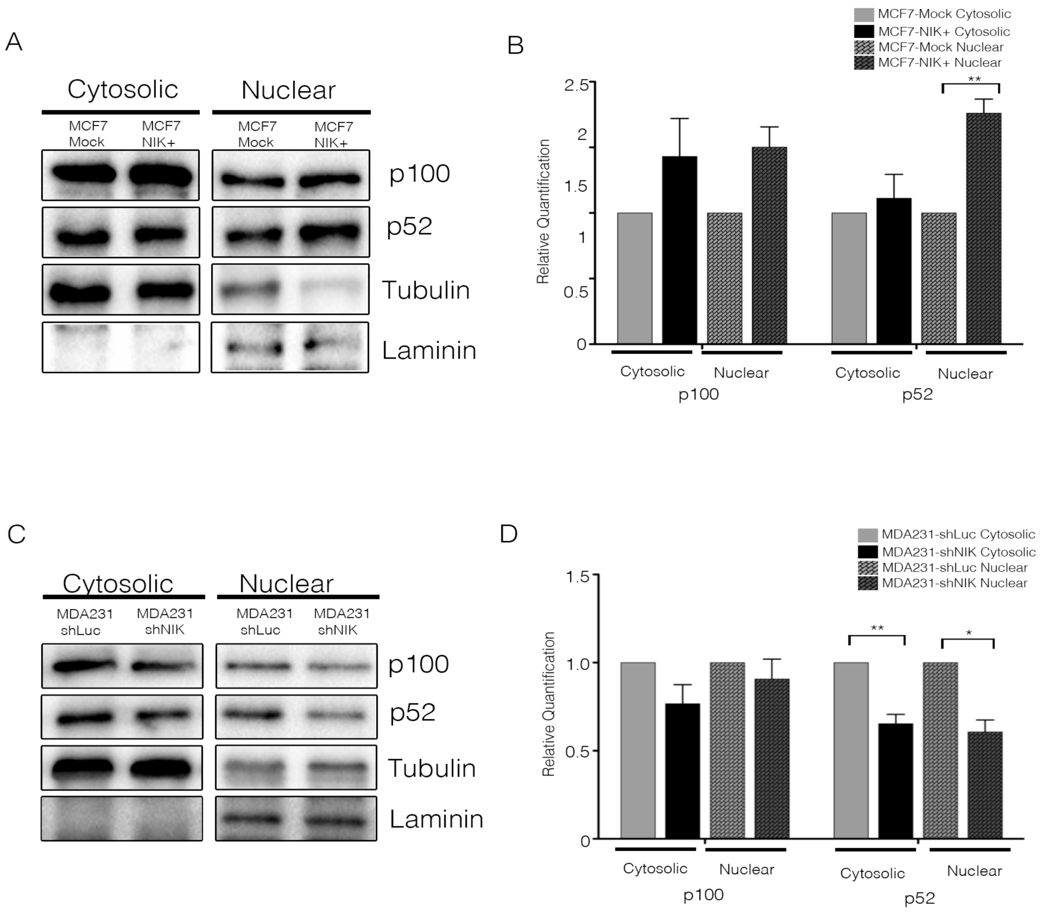

SUPPLEMENTARY FIGURE 4. NIK promotes the processing of p100 to p52. (A): Subcellular fraction was carried out with $1x10^6$ MCF7 NIK-overexpressing or MCF7 mock cells. Western Blot of p100 and p52 was performed in nuclear and cytosolic fraccion. (B) Densitometric scans from three independent assays were quantified, nuclear expression was normalized to lamine A/C and cytosolic expression was normalized to α Tubulin. Data are expressed as fold change relative to mock cells (n=3, error bars are +/- s.e.m, **p=0.002). (B): Subcellular fraction was carried out with $1x10^6$ MDA-MB-231 depleted NIK cells or mock cells. Western Blot of p100 and p52 was performed in nuclear and cytosolic fraccion. (B) Densitometric scans from three independent assays were quantified, nuclear expression was normalized to lamine A/C and cytosolic expression was normalized to α Tubulin. Data are expressed as fold change relative to mock cells (n=3, error bars are +/- s.e.m, **p=0.0027 and *p=0.02).

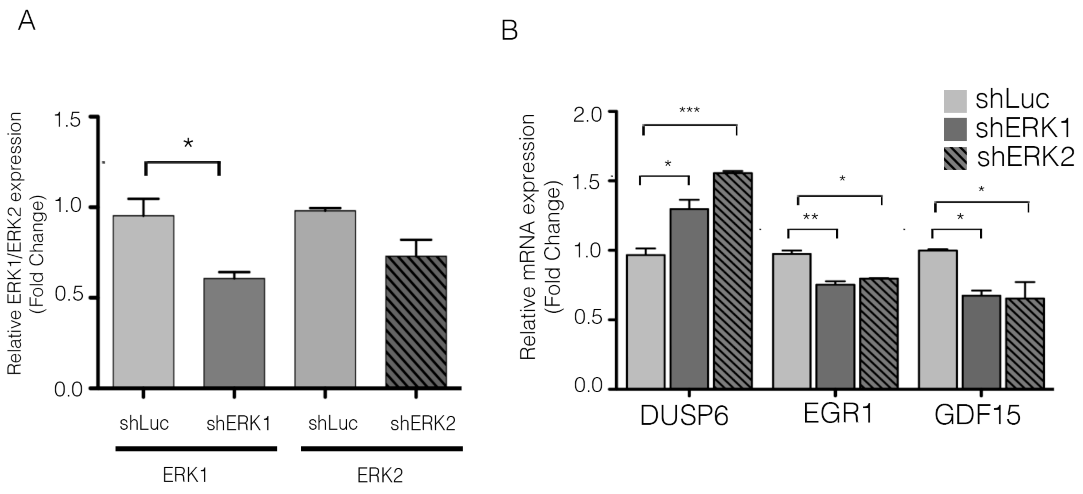

SUPPLEMENTARY FIGURE 5. (A): To knockdown ERK1 and ERK2, two shRNAs against ERK1 and ERK2 were used in MCF7 cell lines. ERK1/2 expression was analyzed by Real Time PCR (RT-qPCR) and normalized to TBP. Data are expressed as fold change relative to mock cells (n=3, error bars are +/- s.e.m, *P=0.02). (B): Expression of DUSP6, EGR1 and GDF15 analyzed by RT-qPCR and normalized to TBP. Data are expressed as fold change relative to mock cells (n=3, error bars are +/- s.e.m, *P<0.05).
